# Supplementary material for: The HVEM-BTLA Axis Restrains T Cell Help to Germinal Center B Cells and Functions as a Cell-Extrinsic Suppressor in Lymphomagenesis
Source: Immunity. 2019 Aug 20;51(2):310–323.e7. doi: 10.1016/j.immuni.2019.05.022 (PMC6703922; doi:10.1016/j.immuni.2019.05.022)
Supplement: Document S1. Figures S1–S5 [file mmc1.pdf]

Immunity, Volume *51*

Supplemental Information

The HVEM-BTLA Axis Restrains T Cell Help to Germinal Center B Cells and Functions as a Cell-Extrinsic Suppressor in Lymphomagenesis

Michelle A. Mintz, James H. Felce, Marissa Y. Chou, Viveka Mayya, Ying Xu, Jr-Wen Shui, Jinping An, Zhongmei Li, Alexander Marson, Takaharu Okada, Carl F. Ware, Mitchell Kronenberg, Michael L. Dustin, and Jason G. Cyster

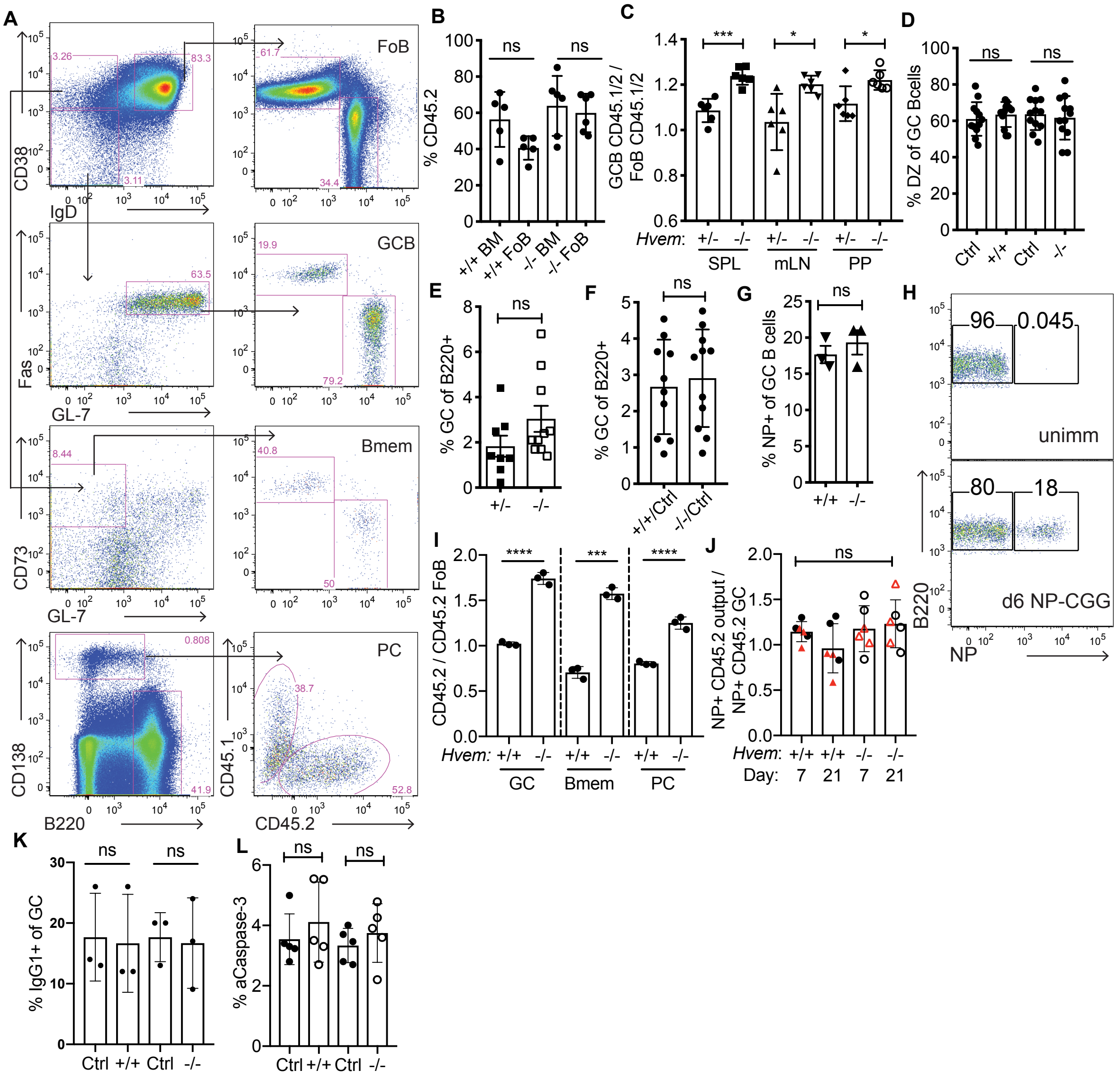

Figure S1. HVEM-deficiency increases GC B cell competitiveness in chronic GCs and GC output after an acute immune response. Related to Figure 1.

(A) Representative flow cytometric analysis of spleen Fo, GC, Bmem, and PC populations from mixed BM chimeras after SRBC immunization. (B) Contribution of CD45.2 cells to $CD93^+$ immature BM B cells and splenic Fo B cells in mixed BM chimeras. Data pooled from 2 experiments. (C) Ratio of CD45.1/2 GC to CD45.1/2 Fo B cells in mixed chimeras made with ~70% of CD45.1/2 *Hvem*$^{+/-}$ or *Hvem*$^{-/-}$ BM with ~30% CD45.1 WT BM in Spleen (SPL), mesenteric LN (mLN) and Peyer's Patches (PP), after intraperitoneal (i.p.) SRBC immunization. Data pooled from 2 independent experiments. (D) Frequency of GC B cells from mixed BM chimeras in dark zone (DZ) state ($CXCR4^{high}CD86^{low}$) after SRBC immunization. Data pooled from 3 experiments. (E) Frequency of GC B cells in full *Hvem*$^{+/-}$ or *Hvem*$^{-/-}$ animals 6–8 days after SRBC immunization. Data pooled from 3 experiments. (F) Frequency of GC B cells in *Hvem*$^{+/+}$ and *Hvem*$^{-/-}$ mixed BM chimeras after SRBC immunization. Data pooled from 3 experiments. (G) Frequency of NP^+ GC B cells in full, non-competitive *Hvem*$^{+/+}$ or *Hvem*$^{-/-}$ chimeras. (H) Representative flow cytometric analysis for splenic NP^+ GC B cell gating on day 6 NP-CGG alum response compared to unimmunized control. (I) Ratio of CD45.2 GC, memory B cells (Bmem), and plasma cells (PC) to CD45.2 Fo B cells at day 8 after SRBC immunization. *Hvem*$^{-/-}$ mixed chimera example shown in panel A. (J) Ratio of CD45.2 NP^+ splenic Bmem (black circles) or PC (red triangles) to CD45.2 NP^+ GC B cells at day 7 and 21 after NP-CGG immunization. Data from 1 experiment and representative of 3 experiments. (K) Frequency $IgG1^+$ of GC B cells in *Hvem*$^{-/-}$ mixed chimeras at day 8 after SRBC. (L) Frequency of anti-active $Caspase\text{-}3^+$ GC B cells from NP-CGG alum immunized mixed BM chimeras day 6-7 directly ex vivo. Data pooled from 2 experiments. 'Ctrl' refers to the respective WT CD45.1 competitor. *$P<0.05$, **$P<0.01$, ***$P<0.001$, ****$P<0.0001$. Unpaired two-tailed Student's t test (B-G, I, K-L), Ordinary One-Way Anova with Bonferroni's multiple comparisons test (J).

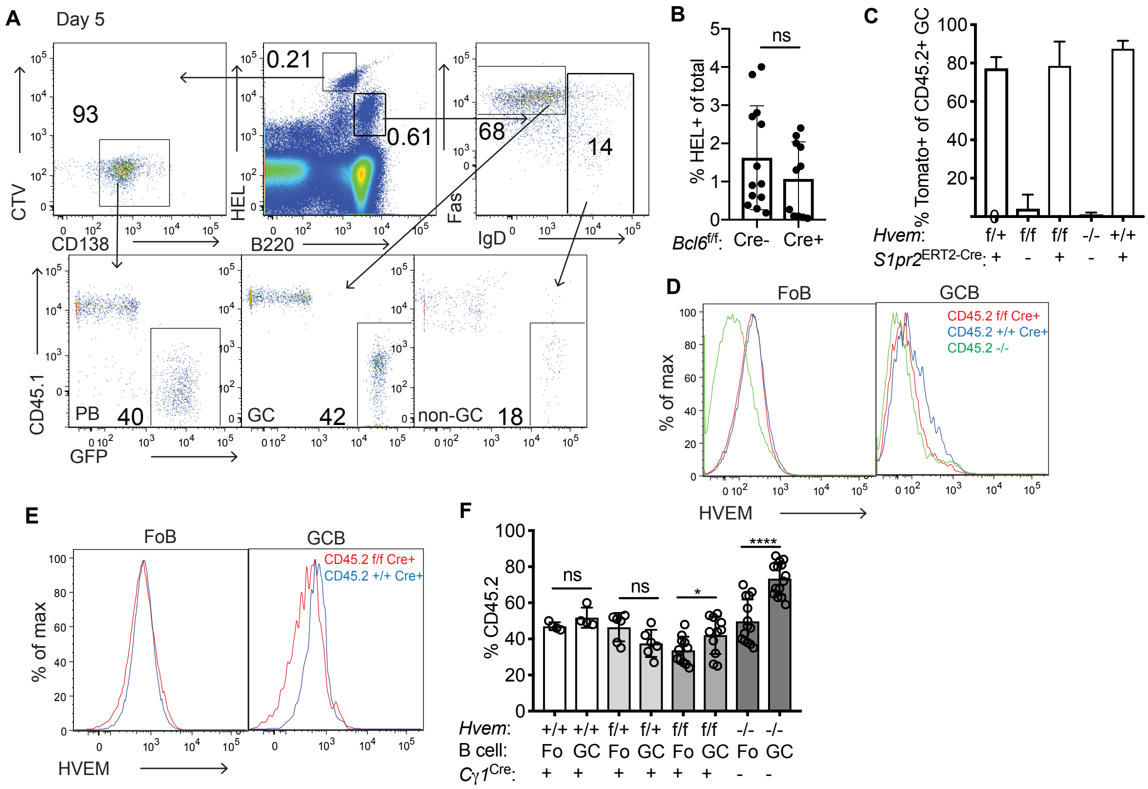

Figure S2. HVEM-deficiency provides B cells with a proliferation advantage early in the response and within the GC. Related to Figure 2.

(A) Representative flow cytometric analysis for gating GFP⁺ HEL⁺ B cells as HEL-intracellular-high CD138⁺ plasmablasts (PB) and HEL-intermediate IgD^{lo}Fas⁺ GC B cells at days 4.5–5. (B) Frequency of HEL⁺ cells of total spleen in *Bcl6*$^{f/f}$ *Cd4*^{Cre} or controls at day 5–7 after 2x-HEL-SRBC. Data pooled from 3 experiments. (C) Frequency of tdTomato⁺ of CD45.2 GC B cells after tamoxifen treatment. Data are pooled from 3 experiments. (D) Representative flow cytometric analysis of HVEM surface levels on Fo and GC B cells in CD45.2 compartment. Red *Hvem*$^{f/f}$ *S1pr2*^{ERT2Cre}, blue *Hvem*$^{+/+}$ *S1pr2*^{ERT2Cre}, and green *Hvem*$^{-/-}$. (E) Representative flow cytometric analysis of HVEM surface levels in *Hvem*$^{f/f}$ *Cγ1*^{Cre} Fo and GC B cells in red and *Hvem*$^{+/+}$ *Cγ1*^{Cre} in blue. (F) *Hvem*$^{f/f}$ *Cγ1*^{Cre} and respective control CD45.2 mixed BM chimeras immunized with NP-CGG and analyzed day 10–13 for the frequency of CD45.2 cells in the Fo and GC compartments. Data are pooled from 3 experiments. *P<0.05, ***P<0.001, ****P<0.0001. Unpaired two-tailed Student's t test (B, F).

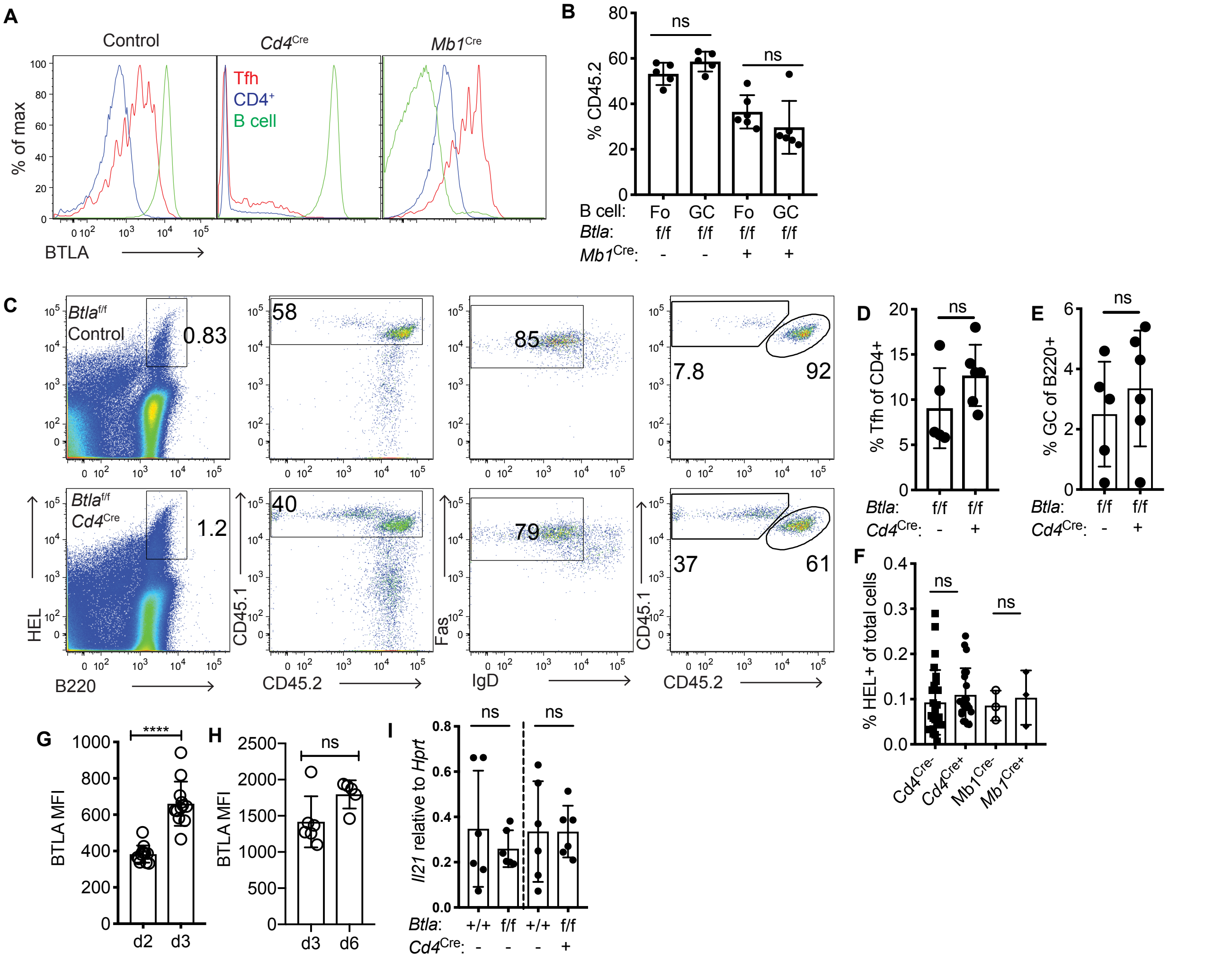

Figure S3. Conditional deletion of BTLA in T cells abrogates HVEM-deficient GC advantage. Related to Figure 4.

(A) BTLA MFI B220^{+} B cells (green), CD4^{+}TCRβ^{+}CXCR5^{+}PD-1^{+} Tfh cells (red), and CD4^{+}TCRβ^{+}CXCR5^{-}PD1^{-} non-Tfh CD4^{+} T cells (blue) in *Btla*$^{f/f}$ animals. Cre^{-} left, *Cd4*^{Cre} middle, and *Mb1*^{Cre} right. (B) Contribution of CD45.2 *Btla*$^{f/f}$ control or BTLA-B cell-deficient *Btla*$^{f/f}$ *Mb1*^{Cre} CD45.2 cells to Fo and GC populations in spleen of mixed BM chimeras made with ~50% CD45.2 and ~50% WT CD45.1 BM at day 7 after SRBC immunization. Data are pooled from 2 experiments. (C) Representative flow cytometric analysis of the frequency of *Hvem*$^{-/-}$ CD45.1/2 HEL^{+} GC B cells in *Btla*$^{f/f}$ control (top) and *Cd4*^{Cre} (bottom) recipients at day 6 after 2x-HEL-SRBC. (D) Frequency of Tfh cells of CD4^{+} T cells in *Btla*$^{f/f}$ *Cd4*^{Cre} animals in the spleen after SRBC. Data are pooled from 2 experiments. (E) Frequency of GC B cells of B cells in *Btla*$^{f/f}$ *Cd4*^{Cre} animals in the spleen after SRBC. Data are pooled from 2 experiments. (F) Frequency of HEL^{+} B cells of total splenocytes after Hy10 transfer and 2x-HEL-SRBC immunization. Data are pooled from 4 experiments. (G) BTLA MFI on OT-II T cells at 48 hr and 72 hr after HEL-OVA or DEL-OVA immunization. Data are pooled from 2 experiments. (H) BTLA MFI on Tfh cells at 3 days and 6 days after 2x-HEL-SRBC immunization. (I) *Il21* mRNA transcript relative to *Hprt* from sorted splenic Tfh cells from *Btla*$^{f/f}$ control CD45.2 or *Cd4*^{Cre} mixed CD45.1/2 WT BM chimeras at day 7 SRBC immunization. Data are pooled from 2 experiments. ****P<0.0001. Unpaired two-tailed Student's t test (B, D-I).

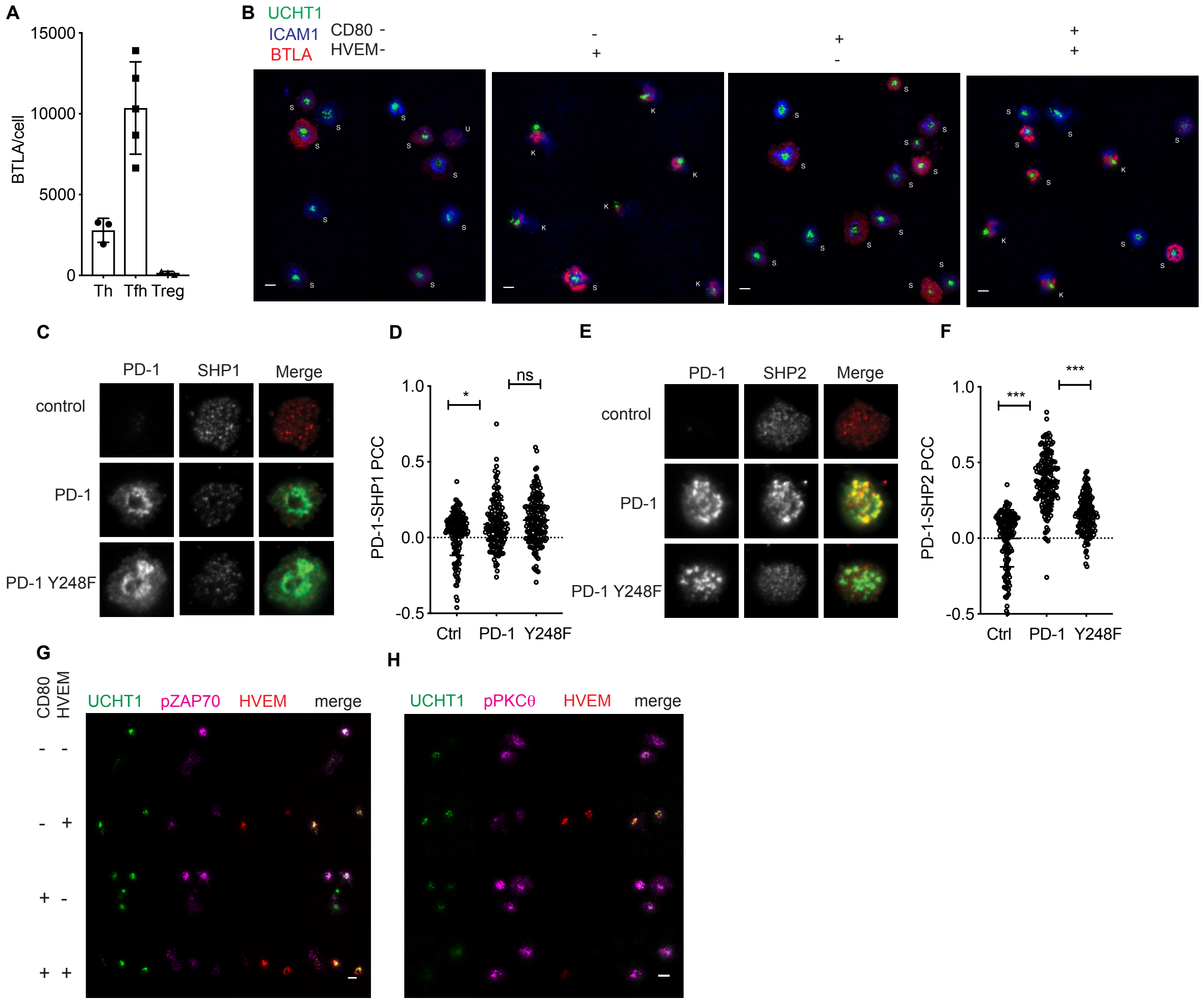

Figure S4. BTLA-HVEM at the immunological synapse recruits SHP1 to inhibit signaling in Tfh cells. Related to Figure 5.

(A) Expression of BTLA on human tonsil Tfh cells, Treg cells and untransfected CD4$^+$ blasts (Th). (B) Representative images of synapse (s) or kinapse (k) states in BTLA-transfected human CD4$^+$ T cell blasts on supported lipid bilayers containing anti-CD3 (UCHT1), ICAM1 and/or HVEM, CD80 as indicated. (C) Representative images of SHP1 recruitment to the synapse in relation to PD-1 or mutant PD-1 Y248F. (D) Quantification of SHP1 colocalization with WT PD-1 or mutant Y248F with or without (Ctrl) PDL-1 in the bilayer. (E) Representative images of SHP2 recruitment to the synapse in relation to PD-1 or mutant PD-1 Y248F. (F) Quantification of SHP2 colocalization with PD-1 or mutant Y248F with or without (Ctrl) PDL-1 in the bilayer. (G) Representative images of p-ZAP70 and (H) p-PKCθ in human CD4$^+$ T cell blasts transfected with BTLA shown at the same LUT. Scale bars are 5 μm. *P<0.05, **P<0.01, ***<P0.001, ****P<0.0001. Mann-Whitney test (D, F.)

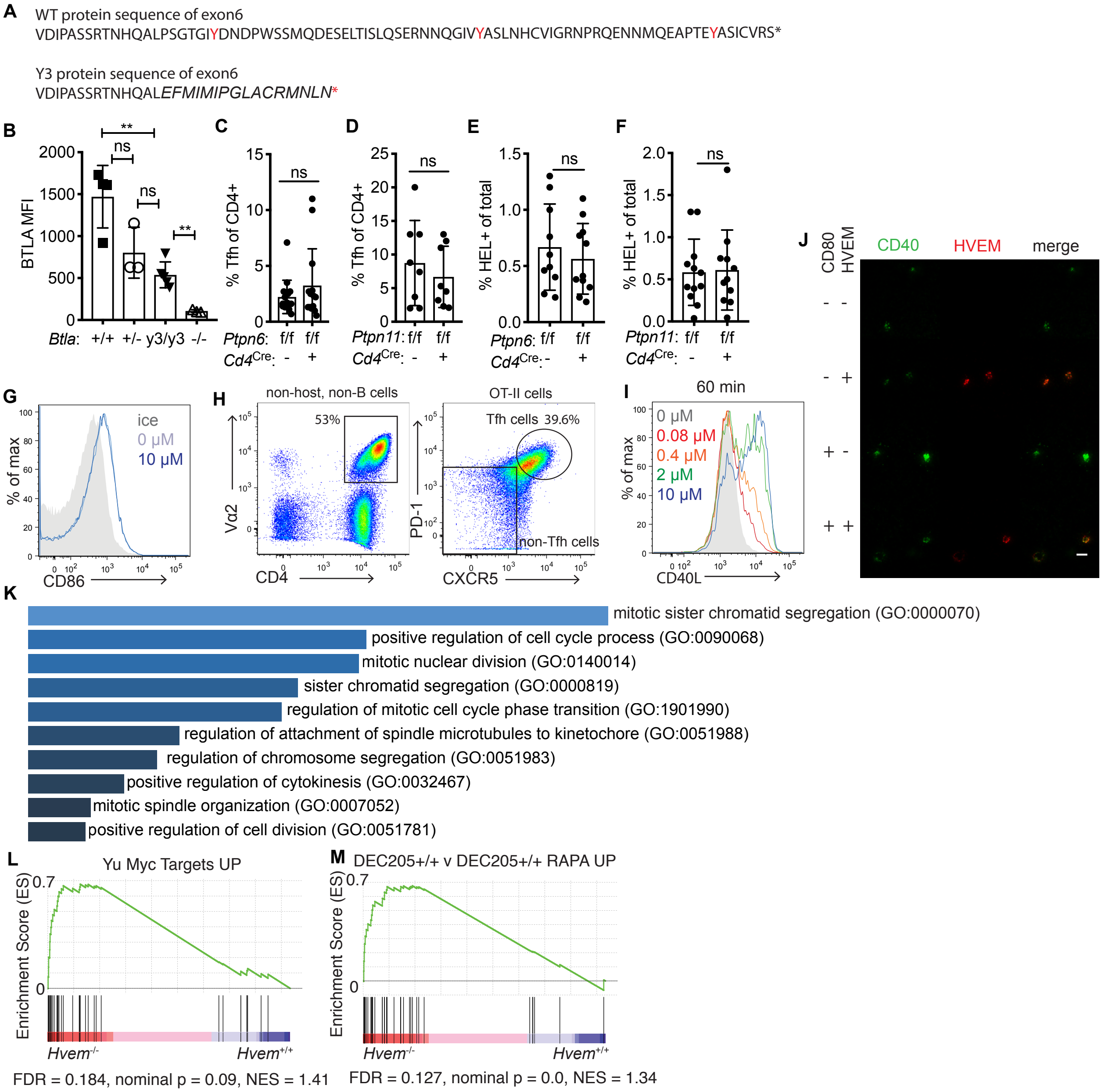

Figure S5. BTLA signaling into the T cell through SHP1 is required for HVEM-deficient GC B cell competitiveness. Related to Figure 6.

(A) Protein sequence of exon 6 of WT BTLA and mutant BTLA Y3. A short out-of-frame deletion was introduced at position 38 of exon 6 using CRISPR mutagenesis in the mouse embryo. Italics indicates nonsense sequence occurring after the deletion. * denotes stop codon. (B) BTLA expression on *Btla*$^{+/+}$, *Btla*$^{+/-}$, *Btlay*$^{3/y3}$, *Btla*$^{-/-}$ Tfh cells. Data pooled from 2 experiments. (C, D) Frequency of Tfh cells in *Ptpn6*$^{f/f}$ (SHP1) (C) and *Ptpn11*$^{f/f}$ (SHP2) (D) control and *Cd4*^{Cre} animals. Data pooled from 2 experiments. (E, F) Frequency of HEL^{+} cells in *Ptpn6*$^{f/f}$ (E) and *Ptpn11*$^{f/f}$ (F) control and *Cd4*^{Cre} animals. Data pooled from 2 experiments. (G) Representative flow cytometric analysis of CD86 surface expression on B cells after a 2 hr pulse with or without OVA peptide (10 μM) and incubation with OT-II Tfh cells for 30 min compared to B cells kept on ice directly ex vivo. (H) Gating strategy for OT-II Tfh cells at d3 after HEL-OVA-SRBC + poly-I:C. (I) Representative flow cytometric analysis of CD40L surface mobilization on OT-II Tfh cells after a 60 min incubation with B cells pulsed with a titration of OVA peptide (0-10 μM). (J) Representative images of CD40 recruitment to the immunological synapse with or without HVEM and CD80 present in the lipid bilayer after 15 min incubation with blasting human CD4^{+} T cells with standardized LUT across panels so fluorescence can be directly compared. Scale bars are 5 μm. (K) Gene ontology (GO) biological processes of genes significantly (padj <0.01) upregulated in *Hvem*$^{+/+}$ v *Hvem*$^{-/-}$ GC B cells at d11 after NP-CGG from RNA-sequencing through Enrichr. Sorted by p-value. (L) GSEA of differentially expressed genes from RNA-sequencing of *Hvem*$^{+/+}$ v *Hvem*$^{-/-}$ mixed BM chimeras at d11 of the NP-CGG alum response compared to Yu Myc targets UP gene set. (M) GSEA of differentially expressed genes from RNA-sequencing of *Hvem*$^{+/+}$ v *Hvem*$^{-/-}$ mixed BM chimeras at d11 of the NP-CGG alum response compared to significant genes (<0.01 padj) with >2 fold increased gene expression in DEC205-WT GC B cells 24 hr after anti-DEC205-OVA compared to DEC205-WT Rapamycin treated. *P<0.05, **P<0.01, ***<P0.001, ****P<0.0001. Unpaired two-tailed Student's test (B-F).
