# Supplementary material for: The HVEM-BTLA Axis Restrains T Cell Help to Germinal Center B Cells and Functions as a Cell-Extrinsic Suppressor in Lymphomagenesis
Source: Immunity. 2019 Aug 20;51(2):310–323.e7. doi: 10.1016/j.immuni.2019.05.022 (PMC6703922; doi:10.1016/j.immuni.2019.05.022)
Supplement: Document S2. Article plus Supplemental Information [file mmc2.pdf]

Immunity

Article

The HVEM-BTLA Axis Restrains T Cell Help to Germinal Center B Cells and Functions as a Cell-Extrinsic Suppressor in Lymphomagenesis

Graphical Abstract

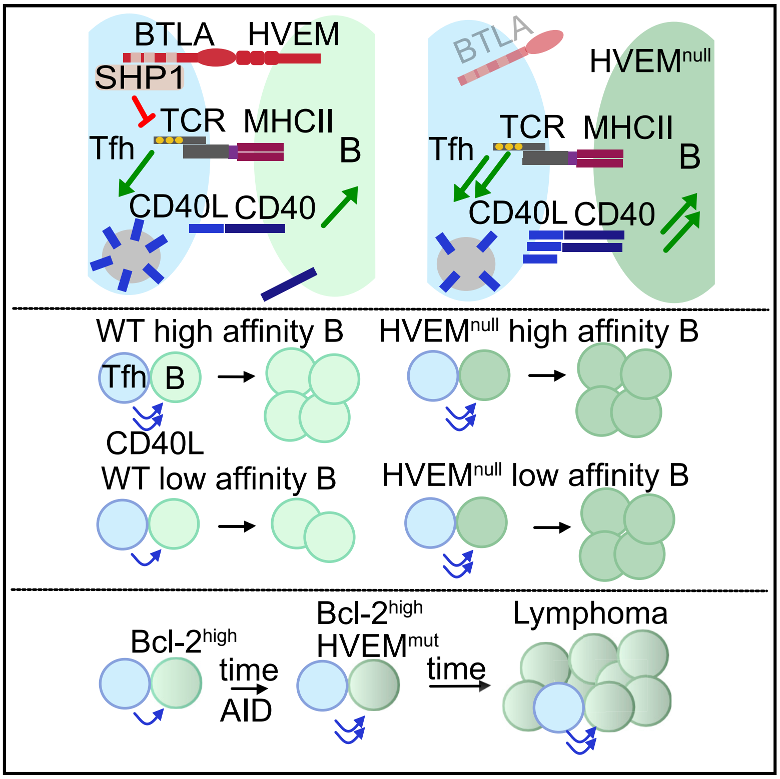

Highlights

- HVEM deficiency increases B cell competitiveness in response to T cell help
- Preformed CD40L upregulation is tuned to TCR signal strength
- HVEM engagement of Tfh BTLA signals via SHP1 to restrain CD40L and B cell proliferation
- T cell BTLA is an extrinsic repressor of Bcl-2-overexpressing GC B cell accumulation

Authors

Michelle A. Mintz, James H. Felce, Marissa Y. Chou, ..., Mitchell Kronenberg, Michael L. Dustin, Jason G. Cyster

Correspondence

jason.cyster@ucsf.edu

In Brief

HVEM is one of the most frequently mutated surface proteins in germinal-center-derived B cell lymphomas. Mintz et al. find that T cell help is restrained by the interaction between HVEM on B cells and BTLA on T follicular helper cells. Loss of the HVEM-BTLA axis leads to increased B cell competitiveness, a reduced stringency of selection, and outgrowth of pre-malignant B cells.

Mintz et al., 2019, Immunity *51*, 310–323
August 20, 2019 © 2019 The Authors. Published by Elsevier Inc.
https://doi.org/10.1016/j.immuni.2019.05.022

CellPress

Cell Press

Immunity
Article

The HVEM-BTLA Axis Restrains T Cell Help to Germinal Center B Cells and Functions as a Cell-Extrinsic Suppressor in Lymphomagenesis

Michelle A. Mintz,[1,2] James H. Felce,[3] Marissa Y. Chou,[1,2] Viveka Mayya,[3] Ying Xu,[1,2] Jr-Wen Shui,[4,7] Jinping An,[1,2] Zhongmei Li,[1] Alexander Marson,[1] Takaharu Okada,[5] Carl F. Ware,[6] Mitchell Kronenberg,[4] Michael L. Dustin,[3] and Jason G. Cyster[1,2,8,*]

[1]Department of Microbiology and Immunology, University of California, San Francisco, San Francisco, CA, USA
[2]Howard Hughes Medical Institute, University of California, San Francisco, San Francisco, CA, USA
[3]Kennedy Institute of Rheumatology, University of Oxford, Oxford, UK
[4]Division of Developmental Immunology, La Jolla Institute for Immunology, La Jolla, CA, USA
[5]RIKEN Center for Integrative Medical Sciences, Yokohama, Kanagawa, Japan
[6]Infectious and Inflammatory Diseases Center, Sanford Burnham Prebys Medical Discovery Institute, La Jolla, CA, USA
[7]Present address: Institute of Biomedical Sciences, Academia Sinica, Taipei, Taiwan
[8]Lead Contact
*Correspondence: jason.cyster@ucsf.edu
https://doi.org/10.1016/j.immuni.2019.05.022

SUMMARY

The tumor necrosis factor receptor superfamily member HVEM is one of the most frequently mutated surface proteins in germinal center (GC)-derived B cell lymphomas. We found that HVEM deficiency increased B cell competitiveness during pre-GC and GC responses. The immunoglobulin (Ig) superfamily protein BTLA regulated HVEM-expressing B cell responses independently of B-cell-intrinsic signaling via HVEM or BTLA. BTLA signaling into T cells through the phosphatase SHP1 reduced T cell receptor (TCR) signaling and preformed CD40 ligand mobilization to the immunological synapse, thus diminishing the help delivered to B cells. Moreover, T cell deficiency in BTLA cooperated with B cell Bcl-2 overexpression, leading to GC B cell outgrowth. These results establish that HVEM restrains the T helper signals delivered to B cells to influence GC selection outcomes, and they suggest that BTLA functions as a cell-extrinsic suppressor of GC B cell lymphomagenesis.

INTRODUCTION

High-affinity germinal center (GC)-derived antibodies are crucial for protection from many pathogens. During a T-cell-dependent response, antigen-reactive B cells are selected for entry into the GC by $CD4^+$ T cells. The earliest T cell selection of the B cell occurs at the T-B border 1–2 days after antigen exposure. After 3–6 days, T cells residing in the GC, termed T follicular helper (Tfh) cells, are required to select GC B cells. Most data favor a model for high-affinity B cell selection where cells with improved affinity internalize and present more of the antigen and win out in receiving more or better-quality T cell help through CD40 ligand (CD40L) or other signals (Bannard and Cyster, 2017; Mesin et al., 2016). However, which factors beyond major histocompatibility complex (MHC) class II-peptide amounts determine the quantity and quality of help delivered to GC B cells is incompletely understood.

The tumor necrosis factor (TNF) receptor superfamily member HVEM (encoded by the gene *TNFRSF14*) is the most highly mutated surface molecule in GC-derived follicular lymphoma (FL) and diffuse large B cell lymphoma (DLBCL) (Cheung et al., 2010; Lackraj et al., 2018; Launay et al., 2012; Manso et al., 2017; Schmitz et al., 2018). HVEM is expressed in B cells as well as several other cell types and has multiple ligands. Two ligands, LIGHT and LTα3, are members of the TNF superfamily, whereas two other ligands, BTLA and CD160, are members of the immunoglobulin (Ig) superfamily. BTLA and CD160 bind HVEM's first cysteine-rich domain (CRD1), whereas LIGHT binds CRD2 (Ward-Kavanagh et al., 2016). HVEM contains TRAF-binding motifs in its cytoplasmic tail, and cross-linking of HVEM can lead to downstream signaling via NFκB (Cheung et al., 2009b; Hsu et al., 1997; Shui et al., 2012). In addition to acting as a receptor and transducing intracellular signals, HVEM can act as a ligand and transmit signals into BTLA-expressing cells (Sedy et al., 2005).

BTLA is widely expressed by immune cells and is highly expressed on B cells, dendritic cells (DCs), and some effector T cells (Murphy et al., 2006). BTLA contains ITIM (immunoreceptor tyrosine-based inhibition) motifs in its cytoplasmic domain and can recruit both SH2-domain-containing tyrosine phosphatases SHP1 and SHP2, but not SHIP or SAP (Chemnitz et al., 2004; Gavrieli et al., 2003; Steinberg et al., 2011; Watanabe et al., 2003). BTLA can negatively regulate both T cell receptor (TCR) and B cell receptor (BCR) signaling *in vitro* (Vendel et al., 2009; Wu et al., 2007) and can be recruited to the immune cell interface (Owada et al., 2010). BTLA also contains a Grb2 binding site that might promote $CD8^+$ T cell cytokine production and proliferation (Ritthipichai et al., 2017; Wakamatsu et al., 2013).

 © 2019 The Authors. Published by Elsevier Inc.
This is an open access article under the CC BY license (http://creativecommons.org/licenses/by/4.0/).

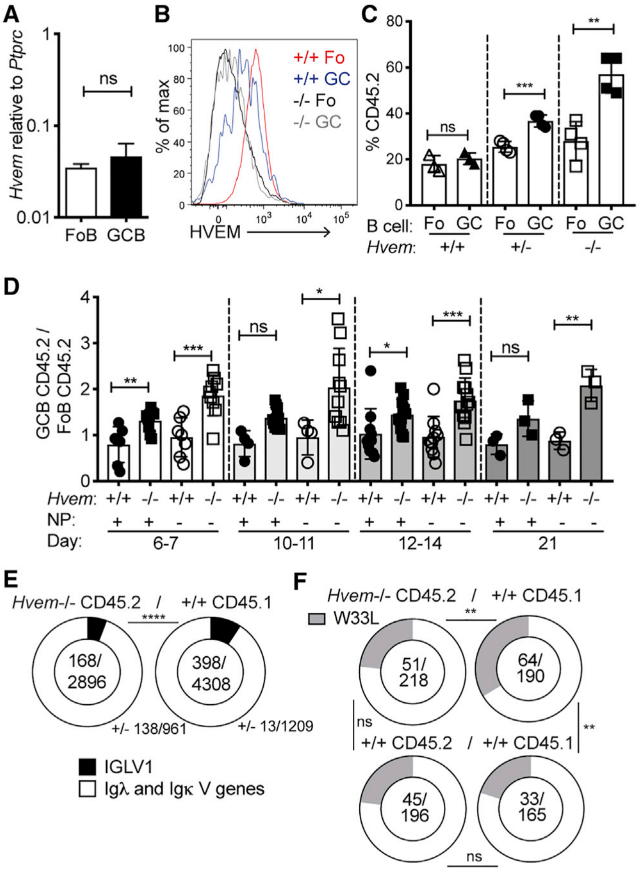

Figure 1. HVEM Deficiency Increases GC B Cell Competitiveness

(A) Quantitative PCR analysis of *Hvem* (*Tnfrsf14*) transcript abundance, relative to *Ptprc* abundance, in follicular (Fo) and GC B cells.

(B) Representative flow-cytometric analysis of HVEM surface expression on $Hvem^{+/+}$ Fo and GC B cells compared with $Hvem^{-/-}$ cells.

(C) Contribution of $Hvem^{+/+}$, $Hvem^{+/-}$, and $Hvem^{-/-}$ CD45.2 cells to Fo and GC B cell populations in the spleen of mixed BM chimeras made with ~30% CD45.2 and ~70% WT CD45.1 BM at day 8 after SRBC immunization. Data are representative of more than three independent experiments.

(D) Ratio of $Hvem^{+/+}$ or $Hvem^{-/-}$ NP^+ or NP^- CD45.2 GC cells to total CD45.2 Fo B cells in mixed BM chimeras after NP-CGG alum immunization at days 6–21 of the splenic response. Data are shown as ratios to simplify varied mixing of ~20%–50% CD45.2 and ~80%–50% CD45.1 BM across experiments. Data are pooled from more than five experiments.

(E) MIXCR BCR repertoire analysis of Ig light-chain V gene usage from paired-end bulk RNA sequencing on fluorescence-activated cell sorting (FACS)-sorted GC B cells from $Hvem^{-/-}$ CD45.2 (n = 3) and WT CD45.1 (n = 3) from the same mixed BM chimeras at day 11 after NP-CGG alum immunization.

(F) Frequency of W33L mutations in the V_H186.2 heavy chain from sorted CD45.2 $Hvem^{+/+}$ or $Hvem^{-/-}$ cells and CD45.1 WT GC B cells at days 11–13 after NP-CGG immunization of mixed BM chimeras. Data are pooled from five experiments.

Unpaired Student's t test (A, C, and D) or chi-square and Fischer's exact test (E and F): *$p < 0.05$, **$p < 0.01$, ***$p < 0.001$, ****$p < 0.0001$. Bars indicate mean ± SD. See also Figure S1.

Therefore, the signaling actions of BTLA might differ between different cell populations and need to be defined on a cell-type-by-cell-type basis.

BTLA is a Tfh cell marker, yet its role in these cells is not well defined (Chtanova et al., 2004; Nurieva et al., 2009). In one study, $Btla^{-/-}$ T cells supported a slightly greater IgG2a and IgG2b response following OVA immunization (Kashiwakuma et al., 2010). In a recent study, small hairpin RNA (shRNA) targeting of *Hvem* in a Bcl-2-driven model of FL increased lymphomagenesis (Boice et al., 2016). Hematopoietic shRNA targeting of *Btla* also increased lymphomagenesis, and the authors suggest that this reflects a function of HVEM within B cells engaging BTLA in B cells to transmit BTLA-mediated BCR-repressive signals. Whether such signals occur in normal GC B cells remains unclear.

Here, we found that HVEM acts to restrain B cell participation in the GC response by signaling via BTLA on T cells. HVEM deficiency provided a proliferative advantage to B cells as early as day 3 or 4 of the response. HVEM engagement of BTLA on T cells decreased TCR signaling and the amount of preformed CD40L mobilized to the T cell surface. Thus, HVEM on B cells restrains T cell help to influence GC selection outcomes, and interfering with this regulatory axis provides a competitive advantage in GC B cell lymphomagenesis.

RESULTS

HVEM Deficiency Increases GC B Cell Competitiveness

Hvem (*Tnfrsf14*) transcripts were expressed at similar levels in follicular and GC B cells (Figure 1A), and HVEM was present on the surface of both cell types, though at lower levels on GC B cells (Figure 1B). To test whether HVEM is an intrinsic regulator of GC B cell responses, we generated mixed-bone-marrow (BM) chimeric mice where BM from wild-type (WT) CD45.1 donor mice was mixed with CD45.2 BM from $Hvem^{+/-}$ or $Hvem^{-/-}$ mice. After immunization with sheep red blood cells (SRBCs), HVEM-deficient B cells were represented more in GCs than in the follicular compartment (Figure 1C and Figure S1A). HVEM heterozygosity led to an intermediate phenotype (Figure 1C). The participation of $Hvem^{-/-}$ B cells in the follicular compartment matched that of developing B cells in the BM (Figure S1B). HVEM deficiency also provided GC B cells with increased competitiveness in chronic GCs in the mesenteric lymph node and Peyer's patches (Figure S1C). $Hvem^{-/-}$ B cells were similarly over-represented in the light and dark zones of the GC (Figure S1D). Although HVEM deficiency gave GC B cells a competitive advantage in the mixed setting, it did not lead to a significant increase in GC size in competitive or non-competitive conditions (Figures S1E and S1F).

To permit tracking of antigen-specific B cells during the GC response, we immunized mixed BM chimeras with the haptenated antigen NP-CGG in alum adjuvant. In the GC response at day 6 or 7 after immunization, HVEM-deficient B cells were more frequent in GCs than in the follicular compartment (Figure 1D). Notably, this advantage was more pronounced in the non-hapten binding (NP^-) GC B cells than in the NP^+ fraction (Figure 1D). HVEM-deficient GC B cells maintained approximately the same magnitude of advantage over the course of the GC response through day 21 (Figure 1D). The frequency of NP^+ GC B cells was similar between full $Hvem^{-/-}$ animals and WT littermate controls, suggesting that there is not a baseline repertoire difference (Figures S1G and S1H).

Paired-end RNA sequencing and BCR gene-usage analysis through MIXCR (Bolotin et al., 2015) at day 11 of the response

showed that $Hvem^{-/-}$ GC B cells used *Iglv1*, the canonical λ chain expanded during NP responses in C57BL/6 mice, less than their WT competitors (Figure 1E). We performed PCR on sorted GC B cells from mixed BM chimeras to examine the frequency of high-affinity mutations in the canonical NP-responding gene *VH186.2* at days 11–13. When referenced against the WT mixed chimeras, HVEM-deficient GC B cells had a lower stringency in selection for the W33L mutation than WT GC B cells in the same animal (Figure 1F).

The SRBC-immunized mixed BM chimeras showed more HVEM-deficient than WT memory B (Bmem) cells and plasma cells (PCs) at day 8 (Figures S1A and S1I). HVEM deficiency also increased the frequency of NP^{+} Bmem cells and PCs at days 7–21 of the NP-CGG response (Figure S1J). These data suggest that Bmem cells and PCs were generated approximately in proportion to the GC cells. Additionally, HVEM deficiency did not affect the frequency of $IgG1^{+}$ class-switched B cells in the GC (Figure S1K). One mechanism by which HVEM deficiency could lead to increased GC B cell accumulation is by reduced cell death. However, the frequencies of apoptotic HVEM-deficient and WT GC B cells as measured by active caspase-3 were similar (Figure S1L). These findings led us to consider whether B cell proliferation might be affected.

HVEM Deficiency Provides the Earliest GC B Cells with a Proliferation Advantage

To determine whether HVEM deficiency increases B cell entry into the GC, we crossed the $Hvem^{-/-}$ mouse line to the Hy10 mouse line, which has a BCR specific to hen egg lysozyme (HEL). To test whether $Hvem^{-/-}$ B cells proliferate more than their competitors during the early response, we labeled $Hvem^{-/-}$ and WT Hy10 B cell mixes with cell trace violet (CTV), co-transferred them with OVA-specific OT-II $CD4^{+}$ T cells into WT hosts, and immunized the mice with HEL-OVA or the lower-affinity antigen DEL-OVA (Figure 2A). At day 2, the amount of proliferation in each group was similar, but by day 3, $Hvem^{-/-}$ Hy10 B cells had an increased frequency of cells that had divided greater than four times, as measured by the increase in CTV^{lo} cells (Figures 2B and 2C). The responses to high- and low-affinity forms of the antigen were similar (Figure 2C). When the proliferation rates were referenced against those in the control mixed transfers, the proliferation rate of the WT cells that were in competition with the HVEM-deficient cells appeared decreased (Figure 2C). At days 4.5–5 after immunization, when both plasmablasts (PBs) and GC B cells could be clearly identified by flow cytometry (Figure S2A), $Hvem^{-/-}$ Hy10 B cells were more frequent in the GC compartment than their WT competitors (Figure 2D). There was also an increase in the PB compartment, though this effect was often not as marked as for the GC B cells in the same animals (Figure 2D). These data suggest that HVEM deficiency reveals itself in a competitive environment by allowing $Hvem^{-/-}$ B cells to outcompete the ability of WT B cells to proliferate.

Loss of HVEM in an Ongoing GC Provides B Cells with a Competitive Advantage

Tfh cells are involved in supporting the early steps in GC induction as well as the mature GC response. To test whether the growth advantage of HVEM-deficient B cells requires Tfh cells, we transferred $Hvem^{-/-}$ and WT Hy10 B cell mixtures into Tfh-cell-deficient $Bcl6^{f/f}$ $Cd4^{Cre}$ hosts. At days 5–7 after immunization with 2×-HEL-SRBC (an intermediate-affinity HEL conjugated to SRBCs), Hy10 B cells expanded in both control and $Bcl6^{f/f}$ $Cd4^{Cre}$ hosts (Figure S2B). Although $Hvem^{-/-}$ Hy10 B cells were more frequent than their WT competitors in control hosts, the HVEM-deficient advantage was lost in the Tfh-cell-deficient hosts (Figure 2E).

To determine whether HVEM deficiency provides B cells with a competitive growth advantage once the cells are within the GC, we crossed *Hvem*-floxed CD45.2 animals to the inducible GC Cre line, $S1pr2^{ERT2Cre}$, and to $tdTomato^{f/f}$ reporter animals. We then made mixed BM chimeras with the *Hvem*-floxed line and respective controls, immunized with NP-CGG and treated with tamoxifen at day 3 to activate Cre in $S1pr2^{+}$ GC B cells (Figure 2F). We found that >70%–80% of CD45.2 GC B cells expressed tdTomato, and HVEM surface expression was decreased to nearly $Hvem^{-/-}$ levels in the GC but was unchanged in follicular B cells (Figures S2C and S2D). Importantly, $Hvem^{f/f}$ $S1pr2^{ERT2Cre}$ B cells were 30%–50% more represented in the GC than in the follicular compartment, whereas the control $Hvem^{f/f}$ Cre^{-} and $Hvem^{+/+}$ Cre^{+} GC B cells were equally represented (Figure 2G). A 1 hr EdU labeling analysis showed that a slightly greater fraction of HVEM-deficient than internal control GC B cells were proliferating (Figure 2H). As another way to test the impact of HVEM loss at the GC stage, we crossed *Hvem*-floxed animals to the $C\gamma1^{Cre}$ line, which acts earlier in the response and avoids the need for tamoxifen. In mixed BM chimeras, $Hvem^{f/f}C\gamma1^{Cre}$ GC B cells had a growth advantage over the control GC B cells (Figures S2E and S2F). These findings indicate that HVEM has a restraining influence on B cells during GC seeding and within the GC.

HVEM Signaling Intrinsic to the B Cell Is Not Required for GC B Cell Suppression

To test whether HVEM signaling into the B cell is required for HVEM-mediated restraint of the GC response, we used a gain-of-function approach. BM-chimeric mice were generated with BM that had been transduced with MSCV-Thy1.1 retrovirus encoding full-length or mutated forms of *Hvem*, and the reconstituted mice were immunized with SRBCs (Figures 3A–3C). After overexpression of WT HVEM, Thy1.1 $reporter^{+}$ GC B cells were 50% less represented than follicular B cells, whereas overexpression of an empty vector did not alter $Thy1.1^{+}$ B cell participation in the GC (Figures 3C and 3D).

To determine whether loss of HVEM's ability to recruit TRAF2/5 affects GC B cell participation, we introduced a point mutation that is known to disrupt TRAF recruitment, E271A, into the HVEM cytoplasmic tail (Hsu et al., 1997). This mutant form of HVEM was expressed on the cell surface and repressed B cell participation in the GC to the same extent as WT HVEM (Figures 3B–3D). To rule out signaling via other parts of the HVEM cytoplasmic tail, we generated a mutant harboring a stop codon at position 235 (Δ235) immediately after the transmembrane domain. This construct suppressed B cell participation in the GC similarly to the WT (Figures 3B–3D). The Δ235 mutant also restrained B cell participation in the GC when expressed in $Hvem^{-/-}$ cells (Figure 3E). These data demonstrate that HVEM's intrinsic

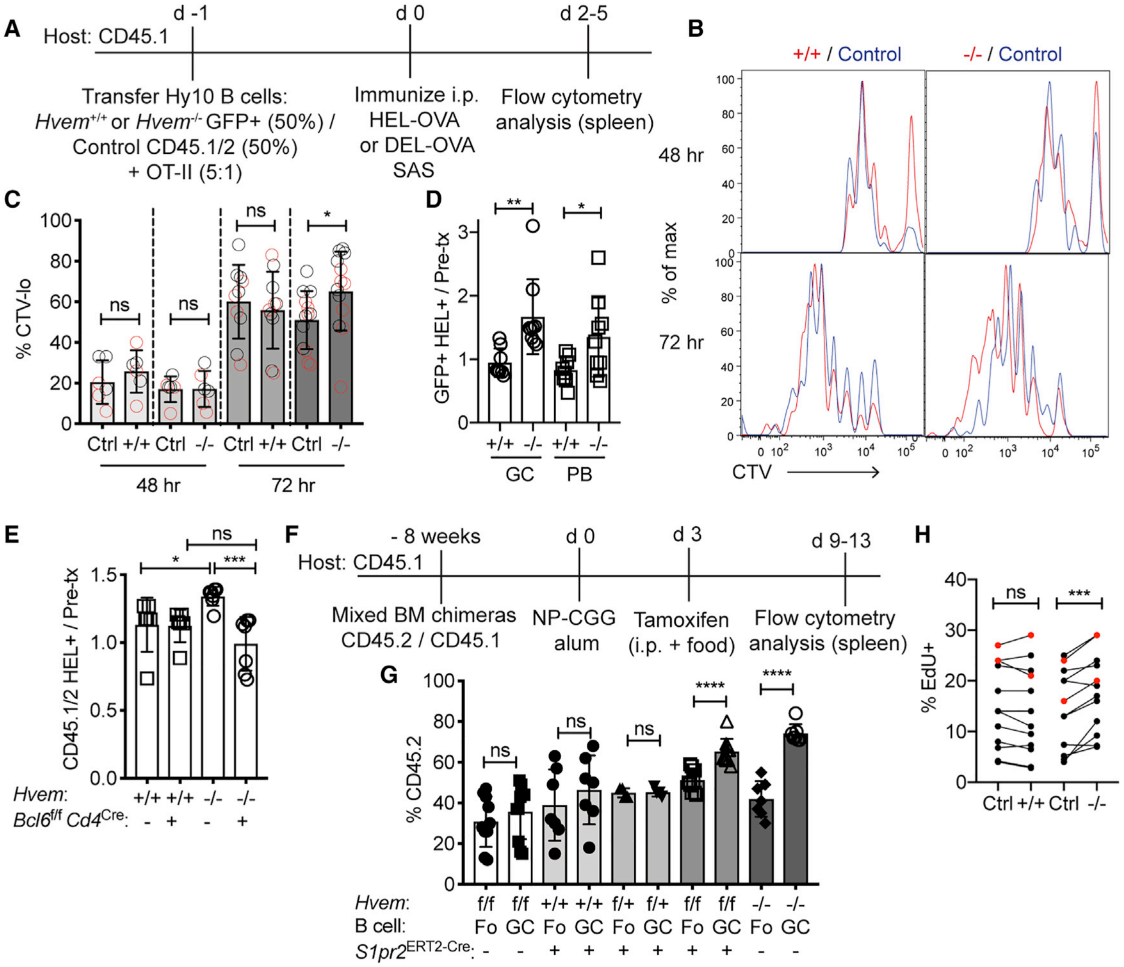

Figure 2. HVEM Deficiency Provides B Cells with a Proliferation Advantage Early in the Response and within the GC

(A) Experimental scheme and timeline for experiments in (B)–(D). CD45.1/2 indicates cells from mice carrying both a CD45.1 and a CD45.2 allele. SAS indicates Sigma Adjuvant System.

(B) Representative CTV dilution at 48 and 72 hr of Hy10 B cells compared with co-transferred WT Hy10 B cells after DEL-OVA immunization. *Hvem*$^{+/+}$ or *Hvem*$^{-/-}$ Hy10 is in red, and WT mixing partner (control) is in blue.

(C) Frequency of CTV-diluted (defined as more than three divisions at 48 hr and more than four divisions at 72 hr) Hy10 B cells compared with their WT mixing controls. Gray circles indicate HEL-OVA immunization, and red circles indciate DEL-OVA immunization. Data are pooled from three experiments.

(D) Ratio of the frequency of GFP$^+$HEL$^+$ GC B cells or PBs at days 4.5–5 after HEL-OVA immunization to the pre-transfer frequency of GFP$^+$HEL$^+$ B cells. Data are pooled from three experiments with pre-transfer mixtures of ~20%–60% GFP$^+$ cells.

(E) Mixed Hy10 transfer with endogenous T cell response into *Bcl6*$^{f/f}$*Cd4*^{Cre} hosts and 2×-HEL SRBC immunization. The ratio of the frequency of CD45.1/2 *Hvem*$^{+/+}$ or *Hvem*$^{-/-}$ Hy10 to the pre-transfer frequency is shown. Tfh-cell-deficient (*Bcl6*$^{f/f}$*Cd4*^{Cre}) and control hosts were analyzed at days 5–7. Data are pooled from three experiments.

(F) Experimental scheme and timeline for experiments in (G).

(G) Frequency of CD45.2 Fo and GC B cells in the tamoxifen-treated mixed BM chimeras. Data are pooled from three experiments and include chimeras with 10%–60% CD45.2 Fo B cells.

(H) Frequency of EdU$^+$IgD^{lo}EphrinB1$^+$ GC B cells in *Hvem*$^{+/+}$ and *Hvem*$^{-/-}$ mixed BM chimeras immunized with NP-CGG. Animals were treated with EdU 1 hr before analysis at day 6 or 7 (black circles) and day 21 (red circles). Data are pooled from three experiments.

Unpaired two-tailed Student's t test (C–E and G) or paired two-tailed Student's t test (H): *$p < 0.05$, **$p < 0.01$, ***$p < 0.001$, ****$p < 0.0001$. See also Figure S2.

signaling into the B cell is not required for its ability to restrain B cell participation in the GC response.

A point mutation at Y61A within the HVEM CRD1 disrupts binding to BTLA and CD160 (Cheung et al., 2009a). Unlike WT HVEM, Y61A HVEM failed to reduce the participation of transduced B cells in the GC response, suggesting that HVEM's ability to bind a ligand through CRD1 is required (Figures 3F and 3G).

BTLA on CD4$^+$ T Cells Is Required for HVEM-Deficient GC B Cell Competitiveness

Given that BTLA is an inhibitory receptor highly expressed in the GC on both B cells and T cells (Figure 4A), we tested whether BTLA is required for HVEM's suppressive ability. When HVEM was overexpressed in *Btla*$^{-/-}$ BM-chimeric mice, Thy1.1 reporter$^+$ GC B cells participated in the GC at the same frequency as in follicular B cells (Figure 4B). This finding contrasts

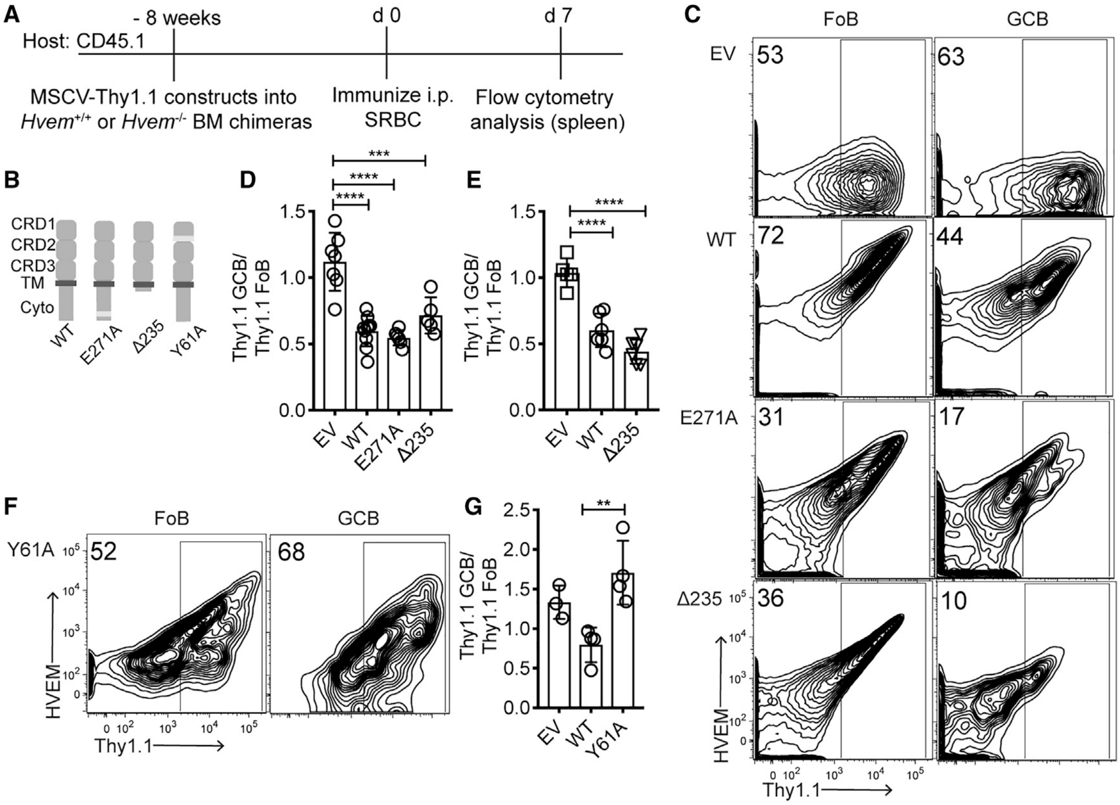

Figure 3. HVEM Signaling Intrinsic to the B Cell Is Not Required for GC B Cell Suppression

(A) Experimental scheme and timeline for experiments in (B)–(G).

(B) Model of HVEM WT, E271A (TRAF2/5 binding mutant), Δ235 (truncation of cytoplasmic tail), and Y61A (CRD1 binding mutant).

(C) Representative flow-cytometric analysis of HVEM and Thy1.1 expression on Fo and GC B cells in BM chimeras transduced with empty vector (EV), HVEM WT, HVEM E271A, and HVEM Δ235.

(D) Frequency of Thy1.1$^+$ cells in GC versus Fo in *Hvem*$^{+/+}$ BM-reconstituted chimeras. Data are pooled from three experiments.

(E) Frequency of Thy1.1$^+$ cells in GC versus Fo in *Hvem*$^{-/-}$ BM-reconstituted chimeras. Data are pooled from two experiments.

(F) Representative flow-cytometric analysis of HVEM and Thy1.1 expression on Fo and GC B cells in HVEM-Y61A-transduced BM chimeras.

(G) Frequency of Thy1.1$^+$ cells in GC versus Fo in *Hvem*$^{-/-}$ BM in pLN response. Data are pooled from two experiments.

Ordinary one-way ANOVA with Bonferroni's multiple-comparisons test (D, E, and G): *$p < 0.05$, **$p < 0.01$, ***$p < 0.001$, ****$p < 0.0001$.

with the repressive effect of HVEM overexpression on B cells in WT BM-chimeric mice (Figure 3D) and suggests that BTLA is the relevant ligand for HVEM's ability to suppress participation in the GC.

In vitro studies have shown that BTLA in B cells can inhibit BCR signaling, and it has been suggested that HVEM-BTLA can interact in *cis* within the same cell (Cheung et al., 2009a; Vendel et al., 2009), and a role for such *cis* interactions has been invoked to explain findings in a mouse FL model (Boice et al., 2016; Huet et al., 2018; Verdière et al., 2018). If the actions of HVEM in restraining B cell participation in the GC were dependent on *cis* engagement with BTLA, B cells lacking BTLA would be predicted to have a GC growth advantage similar to that observed for HVEM-deficient cells. Analysis of mice reconstituted with a mixture of WT and *Btla*$^{-/-}$ BM showed that cell-intrinsic loss of BTLA did not alter the frequency of B cells in the GC (Figure 4C). Similar results were obtained when mixed BM chimeras were made with BM from *Btla*$^{f/f}$ *Mb1*^{Cre} mice that selectively lack BTLA in B cells (Figures S3A and S3B).

To determine whether BTLA on bystander B cells acts in *trans* to influence antigen-reactive B cells, we transferred *Hvem*$^{-/-}$ and WT Hy10 B cell mixes into *Btla*$^{f/f}$ *Mb1*^{Cre} or control hosts that were then immunized with 2×-HEL-SRBC (Figure 4D). *Hvem*$^{-/-}$ Hy10 B cells maintained their growth advantage in the BTLA-B-cell-deficient hosts, suggesting that bystander B cells are not the cellular source of BTLA for HVEM-mediated suppression of GC B cell participation (Figure 4E).

BTLA is a defining marker on Tfh cells compared with other CD4$^+$ T cells (Figure 4A) (Chtanova et al., 2004; Kashiwakuma et al., 2010; Nurieva et al., 2009). To test whether HVEM-deficient GC B cell competitiveness requires BTLA-expressing T cells, we transferred *Hvem*$^{-/-}$ and WT Hy10 B cell mixes into *Btla*$^{f/f}$ *Cd4*^{Cre} hosts that lack BTLA in all T cells (Figure S3A). The hosts were immunized with 2×-HEL-SRBC and analyzed at days 3–6 of the response (Figure 4D). The *Hvem*$^{-/-}$ Hy10 GC B cell growth advantage was abrogated in BTLA-T-cell-deficient mice at all time points analyzed (Figure 4F and Figure S3C). Tfh cells were present at similar frequencies in both

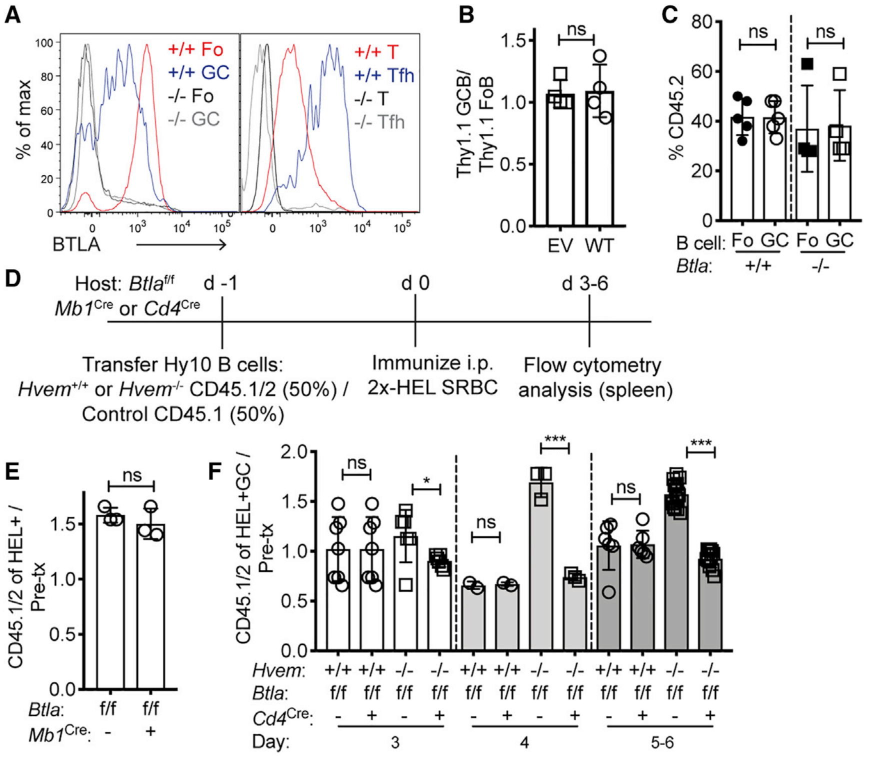

Figure 4. BTLA on T Cells Restrains GC B Cell Competitiveness

(A) Flow-cytometric analysis of BTLA expression on B cells (left) and CD4$^+$ T cells (right) compared with *Btla*$^{-/-}$ cells.

(B) HVEM-overexpressing *Btla*$^{-/-}$ BM chimeras were made by transduction of empty vector (EV) or HVEM (WT) MSCV-Thy1.1 constructs into *Btla*$^{-/-}$ BM and reconstitution of CD4-depleted hosts. Chimeras were then immunized with SRBCs, and the participation of Thy1.1$^+$ cells in GC versus Fo B cells was determined.

(C) Contribution of *Btla*$^{+/+}$ or *Btla*$^{-/-}$ CD45.2 cells to Fo and GC populations in the spleen of mixed BM chimeras made with ~40% CD45.2 and ~60% WT CD45.1 at day 7 after SRBC immunization.

(D) Experimental scheme and timeline for experiments in (E) and (F).

(E) Ratio of the frequency of CD45.1/2 *Hvem*$^{-/-}$ HEL$^+$ B cells in BTLA-B-cell-deficient (*Btla*$^{f/f}$*Mb1*^{Cre}) hosts at day 5 to the pre-transfer frequency.

(F) Ratio of CD45.1/2 *Hvem*$^{+/+}$ or *Hvem*$^{-/-}$ HEL$^+$ GC B cells to the pre-transfer mix in BTLA-T-cell-deficient (*Btla*$^{f/f}$ *Cd4*^{Cre}) hosts at days 3–6. Data are pooled from four experiments.

Unpaired two-tailed Student's t test (B, C, E, and F): *p < 0.05, **p < 0.01, ***<P0.001, ****p < 0.0001. See also Figure S3.

types of recipient animals (Figures S3D and S3E), and the frequency of HEL$^+$ responding B cells was also similar (Figure S3F). BTLA was upregulated in T cells between days 2 and 3 of the response (Figures S3G and S3H), kinetics that matched the timing of when HVEM began to regulate the B cell proliferative response (Figure 2B). These data indicate that BTLA-expressing T cells are required for the *Hvem*$^{-/-}$ GC B cell competitive advantage.

A previous study reported that BTLA deficiency led to increased IL-21 production by *in-vitro*-generated and -restimulated Tfh cells (Kashiwakuma et al., 2010). However, when Tfh cells were generated *in vivo*, we did not observe a difference in *Il21* expression between BTLA-deficient and WT Tfh cells from the same animals (Figure S3I).

BTLA-HVEM at the Immunological Synapse Recruits SHP1 to Inhibit Signaling in Tfh Cells

To determine whether BTLA can alter CD4$^+$ T cell signaling at the immunological synapse when it engages HVEM, we turned to the well-established supported lipid bilayer model (Dustin et al., 2007). Human CD4$^+$ T cell blasts were transfected with BTLA to mimic the surface expression in Tfh cells (Figure S4A) and settled on supported lipid bilayers containing anti-CD3 (UCHT1) and ICAM1 for 15 min before fixation. When HVEM was added to the bilayers, BTLA and HVEM were recruited to the synapse and formed a ring around the central synaptic cleft in cases where a stable synapse had been formed (Figure 5A). In the presence of HVEM, there was a shift in interface organization from the stable synapse to the motile kinapse state (Mayya et al., 2018), where the TCR cluster was at one pole of the interface (Figure 5B and Figure S4B).

BTLA has been shown to recruit SHP1 and SHP2 biochemically through Y257 and Y282 (Gavrieli et al., 2003; Watanabe et al., 2003). BTLA demonstrated strong colocalization with SHP1 at the synapse, whereas SHP2 did not preferentially colocalize (Figures 5C–5F). When the CD4$^+$ T cells blasts were transfected with a BTLA Y257F/Y282F mutant, SHP1 colocalization was diminished and SHP2 was unchanged (Figures 5C–5F). The incomplete effect on SHP1 recruitment most likely reflects the activity of endogenous BTLA in the blasts. These results contrast with the PD-1 molecule that colocalizes preferentially with SHP2 at the synapse (Figures S4C–S4F).

In accord with SHP1 recruitment, inclusion of HVEM in the bilayer inhibited the amount of signaling downstream of the TCR, as measured by pZAP70 and pPKCθ (Figures 5G–5J and Figures S4G and S4H). This was observed for both BTLA-transfected CD4$^+$ T cells and human tonsil-derived Tfh cells (Figures 5G–5J and Figures S4G and S4H). Inclusion of the CD28 ligand CD80 in the bilayer led to an elevated amount of signaling, and HVEM continued to reduce, in most cases significantly, the extent of ZAP70 and PKCθ activation (Figures 5G–5J and Figures S4G and S4H). These data suggest that HVEM engagement of BTLA inhibits T cell activation through SHP1 at the immunological synapse.

BTLA Signaling into the T Cell through SHP1 Is Required for HVEM-Deficient GC B Cell Competitiveness

To test whether BTLA signaling restrains the help provided to B cells, we generated a mutant mouse line (termed BTLA Y3) that lacks the BTLA cytoplasmic tail (Figure 6A and Figure S5A). *Hvem*$^{-/-}$ Hy10 spleen cell mixes were then transferred into *Btla*$^{+/+}$, *Btla*$^{y3/y3}$, and *Btla*$^{-/-}$ hosts, and the mice

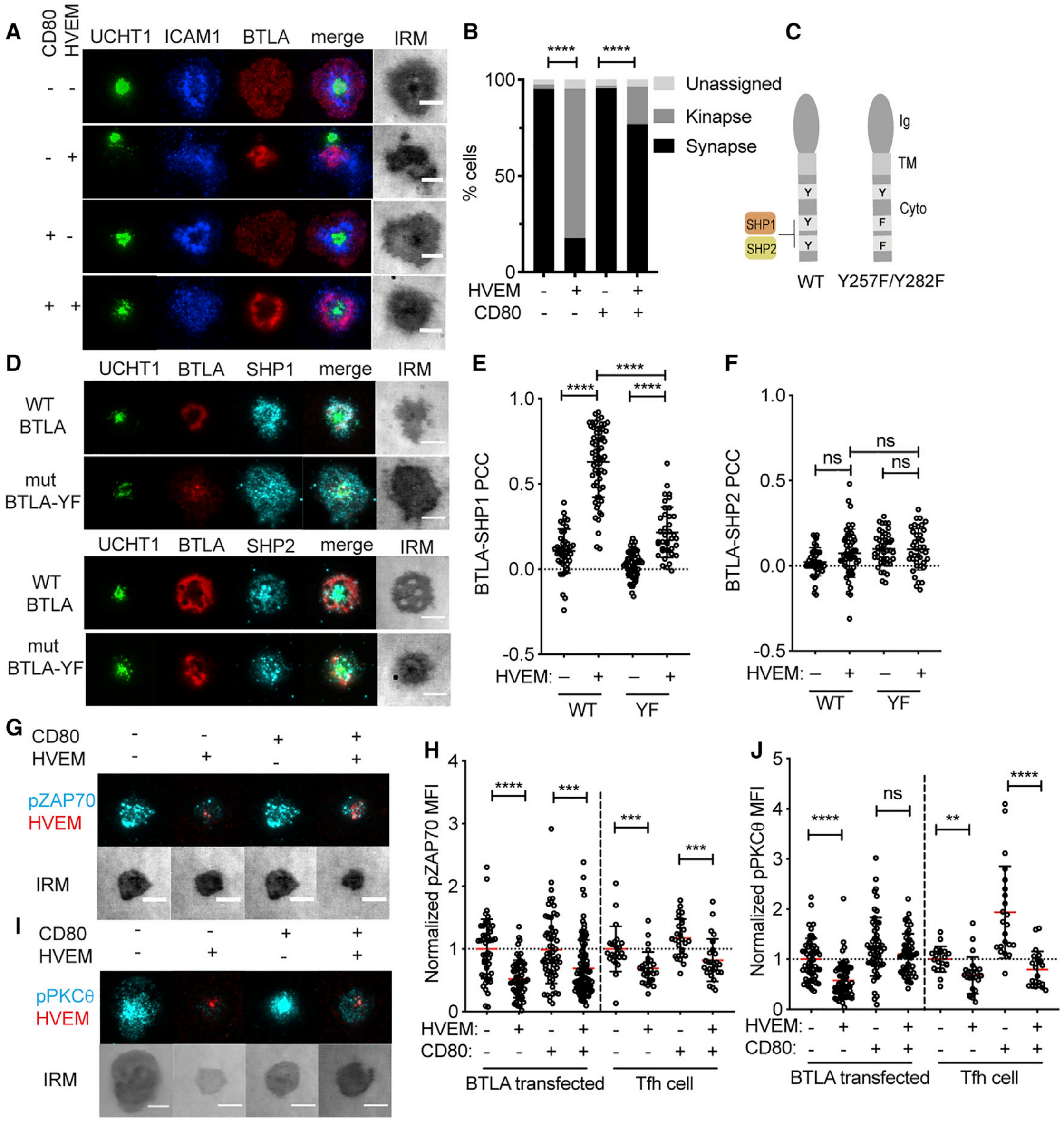

Figure 5. BTLA-HVEM at the Immunological Synapse Recruits SHP1 to Inhibit Signaling in Tfh Cells

(A) Example total internal reflection fluorescence (TIRF) and interference reflection microscopy (IRM) images of BTLA-transfected human CD4$^+$ T cell blasts on supported lipid bilayers containing anti-CD3, ICAM1, and where indicated, HVEM and/or CD80. Staining for CD3 (UCHT1), ICAM1, and BTLA is shown.

(B) Quantification of kinapse versus synapse state of cells imaged as in (A).

(C) Model of human BTLA binding mutant Y257F/Y282F, designed to disrupt SHP1 and SHP2 recruitment to the last two of four cytosolic tyrosines.

(D) Representative images of SHP1 and SHP2 recruitment to the synapse in relation to that of WT or mutant BTLA.

(E and F) Quantification of SHP1 (E) and SHP2 (F) colocalization with WT or mutant BTLA with or without HVEM in the bilayer as determined with Pearson's colocalization coefficient.

(G) Representative images of pZAP70 in human Tfh cells in relation to HVEM in the bilayer after 15 min on the bilayer; a standardized lookup table (LUT) is shown across panels so fluorescence can be directly compared.

(H) Relative amount of pZAP70 in BTLA-transfected CD4$^+$ T cell blasts and Tfh cells with or without HVEM and CD80 in the bilayer (normalized to the condition without HVEM and CD80).

(I) Representative images of pPKCθ in human Tfh cells in relation to HVEM in the bilayer after 15 min on the bilayer; a standardized LUT is shown across panels so fluorescence can be directly compared.

(J) Relative amount of pPKCθ in BTLA-transfected CD4$^+$ T cell blasts and Tfh cells with or without HVEM and CD80 in the bilayer (normalized to the condition without HVEM and CD80). Data are pooled from three independent donors and experiments.

Scale bars: 5 μm. Chi-square and Fischer's exact tests (B) or Mann-Whitney test (E, F, H, and J): *p < 0.05, **p < 0.01, ***p < 0.001, ****p < 0.0001. See also Figure S4.

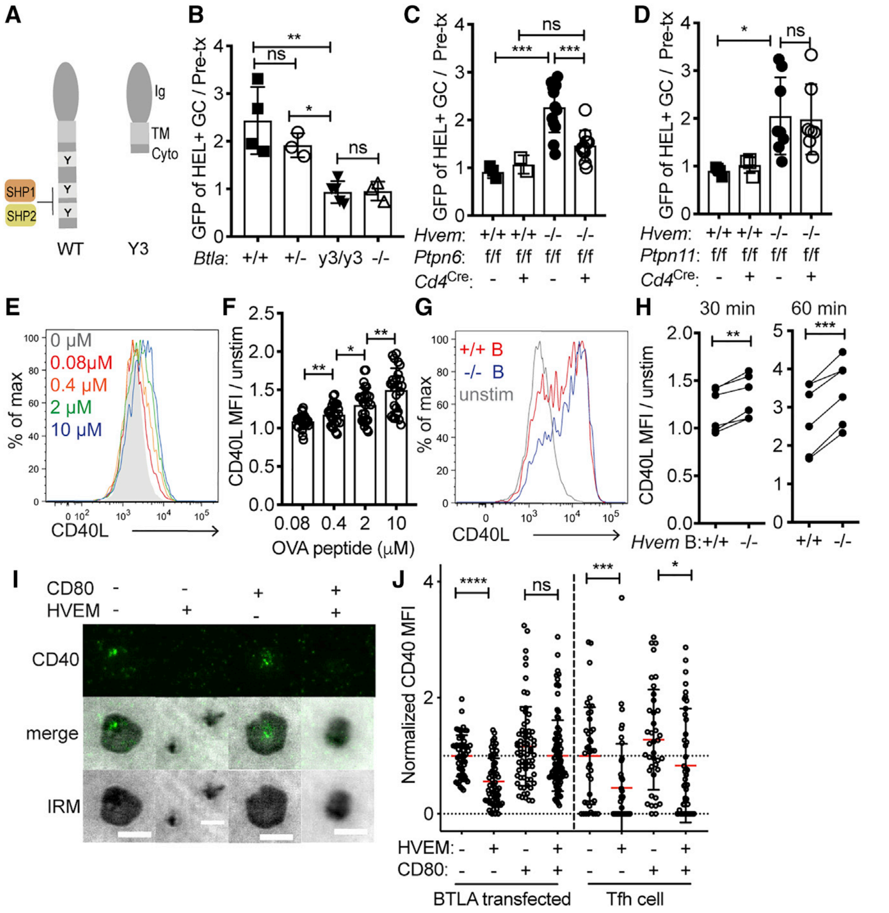

Figure 6. BTLA Signaling into the T Cell through SHP1 Is Required for HVEM-Deficient GC B Cell Competitiveness

(A) Model of murine BTLA truncation, termed Y3, in which all three cytoplasmic tyrosines have been removed.

(B) Ratio of *Hvem*$^{-/-}$ GFP$^+$HEL$^+$ GC (IgD^{lo}Fas$^+$) B cells to the pre-transfer mix (~20% *Hvem*$^{-/-}$ GFP$^+$ Hy10 to ~80% WT CD45.1 Hy10) in *Btla*$^{+/+}$, *Btla*$^{+/-}$, *Btla*$^{y3/y3}$, and *Btla*$^{-/-}$ hosts. Data are pooled from two experiments.

(C) Ratio of *Hvem*$^{+/+}$ or *Hvem*$^{-/-}$ GFP$^+$HEL$^+$ GC B cells to the pre-transfer mix in *Ptpn6*$^{f/f}$ (SHP1) *Cd4*^{Cre} or control hosts. Data are pooled from four experiments.

(D) Ratio of *Hvem*$^{+/+}$ or *Hvem*$^{-/-}$ GFP$^+$HEL$^+$ GC B cells to the pre-transfer mix in *Ptpn11*$^{f/f}$ (SHP2) *Cd4*^{Cre} or control hosts. Data are pooled from three experiments.

(E) Representative flow-cytometric analysis of CD40L surface mobilization on OT-II Tfh cells after a 30 min of incubation; B cells were pulsed with a titration of OVA peptide (0–10 μM).

(F) CD40L expression on OT-II Tfh cells stimulated as in (E) (normalized to expression on unstimulated Tfh cells). Data are pooled from three experiments.

(G) Representative flow-cytometric analysis of CD40L surface mobilization on OT-II Tfh cells after 60 min of stimulation on OVA-peptide-pulsed *Hvem*$^{+/+}$ or *Hvem*$^{-/-}$ B cells.

(H) Normalized CD40L mean fluorescence intensity on OT-II Tfh cells 30 or 60 min after stimulation with OVA-loaded *Hvem*$^{+/+}$ or *Hvem*$^{-/-}$ B cells (2 or 10 μM). Data are pooled from two experiments.

(I) Representative images of CD40 recruitment to the immunological synapse with or without HVEM and CD80 present in the lipid bilayer after 15 min of incubation with human Tfh cells; a standardized LUT is shown across panels so fluorescence can be directly compared. Upper images show immunofluorescence (IF), middle images show overlayed IF and IRM, and lower images show IRM.

(J) Relative CD40 accumulation at synaptic interface in BTLA-transfected CD4$^+$ T cell blasts and Tfh cells with or without HVEM and CD80 in the bilayer (normalized to the condition without HVEM and CD80). Data are pooled from three independent donors and experiments.

Scale bars: 5 μm. Ordinary one-way ANOVA with Bonferroni's multiple-comparisons test (B–D), unpaired two-tailed Student's test (F), paired two-tailed Student's test (H), or Mann-Whitney test (J): *p < 0.05, **p < 0.01, ***p < 0.001, ****p < 0.0001. See also Figure S5.

were immunized with 2×-HEL-SRBC and analyzed at day 5. *Btla*$^{y3/y3}$ hosts phenocopied *Btla*$^{-/-}$ hosts by abrogating the *Hvem*$^{-/-}$ Hy10 GC B cell competitive advantage (Figure 6B). BTLA Y3 mutant protein was less expressed on the T cell surface than on WT Tfh cells (Figure S5B). To control for possible effects of the reduced expression, we also performed transfers into *Btla*$^{+/-}$ recipients whose T cell BTLA expression was similar to that of *Btla*$^{y3/y3}$ mice (Figure S5B). The *Hvem*$^{-/-}$ B cells showed a similar competitive advantage in BTLA heterozygous and WT hosts (Figure 6B). These findings provide evidence that the BTLA cytoplasmic domain in host T cells is required for HVEM-mediated restraint of B cell participation in the GC response.

To test which of BTLA's intracellular binding partners in the T cell restrains the signal to HVEM-expressing GC B cells, we transferred *Hvem*$^{-/-}$ and WT Hy10 B cell mixes into *Ptpn6*$^{fl/fl}$ (SHP1) and *Ptpn11*$^{fl/fl}$ (SHP2) *Cd4*^{Cre} or control hosts. Splenic Tfh cell frequencies in the SHP1 and SHP2 T-cell-deficient hosts were similar to those in controls (Figures S5C and S5D). The *Hvem*$^{-/-}$ GC B cell competitive advantage was lost in SHP1 T-cell-deficient hosts, whereas it was maintained in SHP2 T-cell-deficient hosts (Figures 6C and 6D). The frequency of HEL-binding B cells in both host types was comparable to that in controls (Figures S5E and S5F). These data suggest that after HVEM engagement, BTLA recruits SHP1 to moderate the activation of the T cell and the amount of help provided to the HVEM-expressing B cell.

HVEM-BTLA Interaction Reduces the Amount of CD40-CD40L Brought to the Synaptic Interface

For a Tfh cell to provide greater help to HVEM-deficient B cells than to HVEM-expressing B cells in the same GC microenvironment during interactions that often last only a few minutes, Tfh cell BTLA most likely has to regulate the mobilization of a pre-formed mediator. The best defined pre-formed helper factor in Tfh cells is CD40L (Casamayor-Palleja et al., 1995; Koguchi et al., 2007; 2012). However, it has been unclear whether the amount of preformed CD40L displayed on the T cell surface can be tuned by the strength of the TCR signal. As one approach to test this, we incubated OVA-specific OT-II Tfh cells for 30 min with B cells that had been pulsed with a range of OVA-peptide

concentrations. A 30 min incubation was chosen as a time that allowed cells to interact in the *in vitro* environment while being sufficiently short to ensure that only preformed CD40L could be mobilized. Although the B cells were not intentionally activated, they expressed ICOSL (Xu et al., 2013) and upregulated CD86 during the incubation (Figure S5G), properties that could contribute to their ability to activate Tfh cells. Compared with unstimulated Tfh cell expression of CD40L, B cells presenting increasing amounts of OVA peptide led to an analog upregulation of CD40L on the Tfh cell surface by 30 min, and this was further increased after 60 min (Figures 6E and 6F and Figures S5H and S5I).

We next tested whether HVEM deficiency on OVA-peptide-pulsed B cells influences CD40L upregulation on Tfh cells. Analysis at 30 and 60 min revealed that CD40L exposure was slightly but significantly higher on Tfh cells interacting with HVEM-deficient B cells than on Tfh cells interacting with WT B cells (Figures 6G and 6H). Past studies have shown that CD40 is recruited to the immune synapse in a CD40L-dependent manner (Boisvert et al., 2004; Papa et al., 2017). We therefore returned to the supported lipid bilayer as another approach to test the ability of BTLA to regulate preformed CD40L upregulation. Human CD4^{+} T cell blasts and human Tfh cells were plated on the bilayer containing anti-CD3, ICAM1, CD40, and in some cases CD80. When HVEM was added to the bilayer, the amount of CD40 recruited to the synapse in the 15 min assay was reduced in both the blasts and the Tfh cells (Figures 6I and 6J and Figure S5J). These data suggest that BTLA can restrain the amount of preformed CD40L that is mobilized to the immunological synapse and thus the quality of T cell help.

To examine whether increased T cell help could be read out in the *Hvem*$^{-/-}$ GC B cell as a transcriptional change, we performed RNA sequencing on GC B cells from mixed BM chimeras. The top differentially expressed genes in *Hvem*$^{-/-}$ GC B cells compared with WT GC B cells corresponded to Gene Ontology biological processes for positive regulation of cell-cycle progression (Figure S5K). Previous studies have shown that Tfh cell help signals through CD40 to activate Myc and mTOR1 signaling in GC B cells (Calado et al., 2012; Dominguez-Sola et al., 2012; Ersching et al., 2017; Luo et al., 2018). Gene-set enrichment analysis (GSEA) revealed that *Hvem*$^{-/-}$ GC B cells had more Myc gene signatures than their WT competitors (Figure S5L) (Yu et al., 2005). *Hvem*$^{-/-}$ GC B cells were also enriched with a set of rapamycin-sensitive genes induced downstream of T cell help in GC B cells (Figure S5M) (Ersching et al., 2017). These data suggest that *Hvem*$^{-/-}$ B cells receive more T helper signals than their WT competitors.

BTLA Deficiency in T Cells Leads to GC B Cell Expansion in the Setting of Bcl-2 Overexpression

HVEM mutations in human GC-derived lymphomas often occur in the setting of Bcl-2 overexpression. To determine whether the HVEM-deficient GC growth advantage occurs when B cells constitutively express Bcl-2, we generated *Hvem*$^{-/-}$ *BCL2*-tg mice. Irradiated recipient mice were reconstituted with a mixture of CD45.2 *Hvem*$^{-/-}$ *BCL2*-tg BM and congenically distinguished WT *BCL2*-tg BM, and the chimeric animals were immunized with SRBCs. The HVEM-deficient Bcl-2 GC B cells maintained their growth advantage over WT Bcl-2 competitors (Figure 7A). These data indicate that the growth-promoting pathway mediated by HVEM deficiency is distinct from the pro-survival pathway mediated by Bcl-2.

Bcl-2 is very highly expressed in human FL, and previous work has shown that lines of mice expressing higher amounts of Bcl-2 in B cells than the *BCL2*-tg line have a higher propensity to form lymphomas (Ogilvy et al., 1999). To generate mice with higher expression of Bcl-2 in B cells, we transduced WT or *Hvem*$^{-/-}$ BM with a MSCV-*Bcl2*-Thy1.1 construct. After we reconstituted WT hosts, the Bcl-2-overexpressing BM chimeras generated large splenic GCs in unimmunized mice as early as week 6 after reconstitution, and the GCs increased in size through 14 weeks (Figures 7B and 7C). Compared with WT chimeras, *Hvem*$^{-/-}$ chimeras had increased GC B cell expansion, suggesting that HVEM deficiency cooperates with strong Bcl-2 overexpression.

On the basis of our findings in the preceding sections, we hypothesized that BTLA in the T cell could restrain the amount of help provided to the GC B cell in the setting of Bcl-2 overexpression. To test this possibility, we transduced *Btla*$^{f/f}$ *Cd4*^{Cre} or control BM with the MSCV-*Bcl2*-Thy1.1 construct and used it to reconstitute *Rag2*$^{-/-}$ or *Tcrb*$^{-/-}$ mice. We used T-cell-deficient mice as hosts to ensure an absence of radioresistant WT T cells. When Bcl-2-overexpressing B cells developed in an environment where all the T cells were BTLA deficient, there was increased GC B cell expansion in the spleen (Figures 7D–7F). VH- and VL-region PCR analysis of GC B cells from two of the *Btla*$^{f/f}$ *Cd4*^{Cre} BM chimeras with GC B cell frequencies exceeding 20% of splenic B cells showed evidence of clonal outgrowths (Figure 7G). Clonal dominance was not observable for the light chain, perhaps because even small numbers of polyclonal cells prevent an abundant clone from dominating this PCR reaction. All together, these data indicate that BTLA on the T cell acts as a cell-extrinsic suppressor of Bcl-2-overexpressing GC B cell expansion.

DISCUSSION

The above findings establish that engagement of BTLA on helper T cells by HVEM on B cells signals to restrain B cell expansion during the generation of GC cells and PBs and also acts as a restraint on B cells within the GC. The negative signaling into the T cell depends on the BTLA cytoplasmic domain and on SHP1 recruitment. HVEM engagement of BTLA reduces proximal TCR signaling, and this reduces the output of CD40L and most likely other preformed mediators from the T cell. HVEM thereby restrains B cell proliferation, differentiation, and selection by reducing the delivery of helper signals from the T cell. We also found that the T helper signals restrained by the HVEM-BTLA interaction can exert their growth-promoting effects on Bcl-2-overexpressing (pre-malignant) B cells, providing evidence that BTLA might act as a cell-extrinsic repressor of B cell lymphomagenesis.

Under competitive conditions, proliferation of HVEM-deficient B cells was favored over that of WT B cells, in agreement with other models of B cell competition for T cell help (Gitlin et al., 2014; Schwickert et al., 2011; Zaretsky et al., 2017; Ersching et al., 2017; Yeh et al., 2018). When C57BL/6 mice were deficient in HVEM in all B cells or deficient in BTLA in all T cells, although

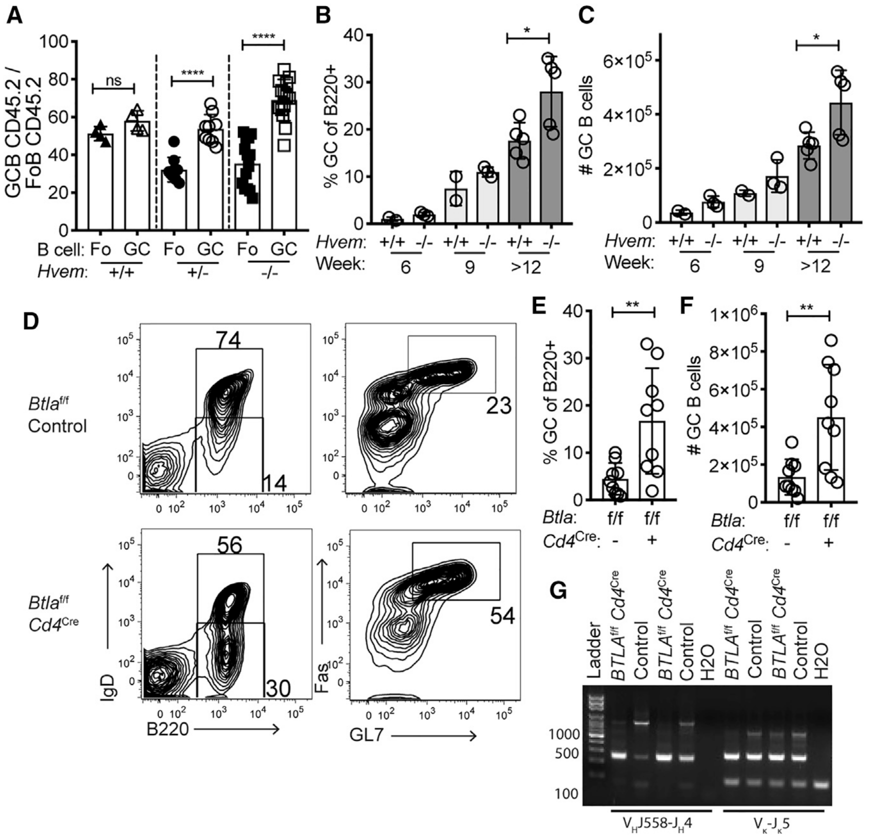

Figure 7. BTLA Deficiency in T Cells Leads to GC B Cell Expansion in the Setting of Bcl-2 Overexpression

(A) Frequency of CD45.2 splenic Fo and GC B cells of Bcl-2 transgenic origin in mixed BM chimeras reconstituted with 30%–50% *Hvem*$^{+/+}$, *Hvem*$^{+/-}$, or *Hvem*$^{-/-}$ Eμ-BCL2-tg CD45.2 BM and 50%–70% WT Eμ-BCL2-tg CD45.1/2 BM at day 7 after SRBC immunization. Data are pooled from three experiments.

(B and C) The frequency (B) and number (C) of spontaneously forming splenic GC B cells in mice reconstituted for 6–14 weeks with *Hvem*$^{+/+}$or *Hvem*$^{-/-}$ BM transduced with MSCV-Bcl2-Thy1.1. Data are pooled from two experiments.

(D) Representative flow-cytometric analysis of spontaneous GC formation in the spleen after 8 weeks of reconstitution with *Btla*$^{f/f}$ or *Btla*$^{f/f}$ *Cd4*^{Cre} BM transduced with MSCV-Bcl2-Thy1.1. Irradiated *Rag2*$^{-/-}$ or *Tcrb*$^{-/-}$ mice were used as hosts.

(E and F) The frequency (E) and number (F) of spontaneously forming splenic GC B cells in BM chimeras from (D). Data are pooled from three experiments.

(G) V_H- and V_L-region PCR analysis of GC B cells from two *Btla*$^{f/f}$*Cd4*^{Cre} chimeras with splenic GC outgrowths > 20% and two *Btla*$^{f/f}$ control chimeras. Unpaired two-tailed Student's test (B,C, E, and F): *p < 0.05, **p < 0.01, ****p < 0.0001.

there was a trend toward increased GC frequencies, the GC response was not significantly enlarged. A study in BALB/c mice reported an enlarged GC response when BTLA was absent, suggesting that the extent of BTLA-mediated regulation of GC B cells might be influenced by background genes (Kashiwakuma et al., 2010). Although we observed a slight increase in *Hvem*$^{-/-}$ GC B cell proliferation in mixed settings across several time points, we did not observe a progressive increase in *Hvem*$^{-/-}$ B cell representation within the GC over time, suggesting there should be increased output of *Hvem*$^{-/-}$ cells from the GC to account for the extra cells generated by proliferation. Our inability to detect more GC B cell death or more Bmem or PC representation than B cell representation in the GC could be because of a small effect size and the possibility that more than one of these output parameters is affected. In addition to leading to increased proliferation of HVEM-deficient cells, lack of HVEM on some B cells appeared to reduce proliferation of competing WT B cells in the same animal. The exact mechanism of the diminished response of the WT cells is unclear, but one possibility is that Tfh cells harbor a limited amount of preformed helper factors (such as CD40L), and when greater amounts are delivered to some cells (*Hvem*$^{-/-}$ in this case), less is available for the other cells present in the same microenvironment. A related possibility, suggested by the propensity of HVEM to convert stable synapses to motile kinapses, is that the increased stability of *Hvem*$^{-/-}$ B cell interactions with Tfh cells diminishes the contact of WT B cells with Tfh cells.

B-T interactions generally occur over periods of minutes, making it unlikely that there is sufficient time for B-cell-triggered induction of new transcription to affect the T cell help delivered during a given contact. Although previous studies have shown that CD40L can be upregulated in a graded manner in proportion to the amount of TCR engagement, these studies were done over periods of 16–20 hr and most likely involved transcriptional induction (Iezzi et al., 2009; Ruedl et al., 2000). In our study, we found that the rapid display of preformed CD40L on Tfh cells occurred in a manner proportional to the amount of MHC peptide on the B cells. In accordance with the fact that stronger signaling into the T cell mobilizes more preformed CD40L, previous work found that co-engagement of ICOS and the TCR enhances CD40L externalization (Liu et al., 2015; Papa et al., 2017). These combined observations suggest that the BTLA-mediated restraint of TCR-induced CD40L mobilization in T cells is due to the negative regulation of proximal TCR signaling.

In biochemical studies, BTLA co-immunoprecipitates with SHP1 and SHP2 (Gavrieli et al., 2003; Watanabe et al., 2003). The molecular basis for the preferential coupling to SHP1 observed here in Tfh cells is not yet known and will need further investigation. These observations contrast with findings for PD-1, which preferentially associates with SHP2 in immune synapse studies with $CD4^+$ and $CD8^+$ T cells (Hui et al., 2017; Yokosuka et al., 2012; this study). However, an *in vivo* study suggests that PD-1 function in effector CD8 T cells is not fully dependent on SHP2 (Rota et al., 2018). Given that PD-1 and BTLA are both highly expressed on Tfh cells, it will be important

to determine whether PD-1 in Tfh cells *in vivo* depends on SHP2 because this could indicate qualitative differences in the type of regulation exerted in Tfh cells by these related checkpoint-family proteins.

HVEM-mediated restraint of the B cell response did not begin immediately, suggesting that some temporal event needs to take place before HVEM-BTLA signaling becomes influential. We speculate that this could at least in part reflect the need for Bcl6 and BTLA upregulation in the activated T cell given that the influence of HVEM deficiency was lost in mice lacking Bcl6 in T cells. It is notable that HVEM surface levels are lower on both mouse and human GC B cells than on naive and early activated B cells. Given the strong growth-repressive effects observed in HVEM gain-of-function experiments, the reduced HVEM levels could be important in permitting transmission of the helper signals needed to support the strong proliferative responses of GC B cells.

Predicting the impact of greater T cell help (indiscriminate of the amount of MHC peptide presented) on the repertoire of B cells selected in the GC is not straightforward. We found that selection of NP-binding B cells was disfavored in HVEM-deficient B cells, perhaps indicating an increased competitiveness of carrier-specific B cells that would normally receive inadequate T cell help and be outcompeted by B cells responding to the readily accessible and highly multivalent NP hapten. Our inability to detect a differential effect of HVEM deficiency on the proliferative response of Hy10 B cells responding to DEL (K_A 10^7 M^{-1}) versus HEL (K_A 10^{10} M^{-1}) (Lavoie et al., 1992) could be because both antigens are in the high-affinity range, and the oligomeric state of the OVA conjugates could further diminish differences in antigen capture and MHC peptide presentation. In the context of NP-based immunizations, a similar increase in representation of non-NP binding B cells has been seen in immunized PD-L1-deficient mice (Shi et al., 2018). The fraction of cells harboring the affinity-improving W33L mutation was lower within the NP-binding *Hvem*$^{-/-}$ GC B cell population than in the internal control WT cell population. It was notable that W33L mutations were more frequent in the WT cells competing with *Hvem*$^{-/-}$ cells than in those competing with other WT cells. One interpretation of these findings is that by diminishing the helper signals available to WT B cells, the HVEM-deficient B cells promote stronger selection for those WT B cells that are able to present the highest amounts of antigen. The combined observations suggest that negative costimulatory molecule signaling in Tfh cells is necessary to maintain the stringency of GC selection. A reduction in B cell selection stringency could explain the autoantibody production that occurs in aged BTLA-deficient 129/SvEV mice (Oya et al., 2008)

In agreement with a previous study on Bcl-2-overexpressing BM chimeras (Boice et al., 2016), in which *Hvem* shRNA-targeted B cells preferentially contributed to GC-derived lymphomas, we found that B cell HVEM deficiency cooperated with Bcl-2 overexpression in promoting GC outgrowth. The authors of the previous study also found that shRNA targeting of *Btla* in hematopoietic cells (HSCs) led to GC lymphomas and attributed this to a mechanism whereby *cis* engagement of BTLA by HVEM leads to BTLA-mediated downregulation of tumor-promoting BCR signals (Boice et al., 2016; Huet et al., 2018; Verdière et al., 2018). In the experiments here, we did not observe any evidence for *cis* or *trans* effects of deleting BTLA from B cells, as measured by participation in the GC response. Instead, we found that deletion of BTLA from T cells led to a growth advantage for HVEM-expressing B cells, including in the setting of Bcl-2 overexpression. Although an important distinction between these studies is that we measured GC participation and Boice et al. measured B cell lymphomagenesis, we believe that the different conclusions can be reconciled. Specifically, Boice et al. did not report enrichment of shRNA-*Btla* B cells over control B cells in BM chimeras, making it seem unlikely that *cis* HVEM-BTLA interaction could be the principle mechanism of HVEM-mediated tumor suppression. Although they also proposed *trans* engagement by HVEM expressed in other B cells (Boice et al., 2016), we believe a more likely explanation for the lymphomas arising in some of the chimeras with shRNA-*Btla* HSCs is that *Btla* transcripts are reduced in the Tfh cell compartment. We believe that our findings together with Boice et al.'s shRNA chimera data support a revised model in which *HVEM* mutation in B cells leads to a loss of negative signaling in Tfh cells and allows *HVEM*-mutant B cells to receive exaggerated helper signals that promote proliferation and accrual of activation-induced cytidine deaminase (AID)-mediated mutations. In this regard, it is notable that FL is known for its rich presence of Tfh cells (de Jong and Fest, 2011; Huet et al., 2018; Verdière et al., 2018). Importantly, the therapeutic benefit of elevating HVEM levels in the FL setting, as Boice et al. (2016) achieved, by using CAR T cells might act by diminishing Tfh cell function and thus support FL cell growth. Future studies are needed to test whether soluble HVEM can reduce Tfh cell function during GC responses and in lymphoma models where B cell HVEM has been mutated.

STAR★METHODS

Detailed methods are provided in the online version of this paper and include the following:

- KEY RESOURCES TABLE
- LEAD CONTACT AND MATERIALS AVAILABILITY
- EXPERIMENTAL MODEL AND SUBJECT DETAILS
  - Mice
- METHOD DETAILS
  - Generation of BTLAY3 Mice
  - Bone Marrow Chimeras
  - Retroviral Constructs and Transductions
  - Cell Isolation and Adoptive Transfer
  - Immunizations
  - Flow Cytometry
  - qPCR and RNA Sequencing
  - GSEA and Gene Ontology
  - NP V_H186.2 Mutation Analysis
  - Tfh Cell CD40L Surface Mobilization
  - Assessment of BCR Clonality by PCR
  - Human CD4$^+$ T Cell Isolation, Stimulation, And Transfection
  - Human Tfh Cell Isolation
  - Quantification of BTLA and HVEM Surface Expression
  - Supported Lipid Bilayer Preparation and Use
  - Immunofluorescence Staining

- ○ TIRF and Confocal Imaging
- ○ Image Analysis
- ○ *In Situ* Analysis of Interaction of SHP1 and SHP2 with PD-1
- ● QUANTIFICATION AND STATISTICAL ANALYSIS
- ● DATA AND CODE AVAILABILITY

SUPPLEMENTAL INFORMATION

Supplemental Information can be found online at https://doi.org/10.1016/j.immuni.2019.05.022.

ACKNOWLEDGMENTS

We thank C. Allen, J. Bluestone, and J. Rubenstein for mice; S. Davis for HVEM-Fc-His and 6×-His PDL1; K. Wucherpfennig for 10×-His-CD58; R. Brink for 2×-HEL; N. Strauli for assistance with running MIXCR; D. Parker and J. Gardell for advice on detecting CD40L; E. Dang, E. Lu, and A. Reboldi for helpful discussions; and M. Meyer-Hermann, O. Bannard, and J. Muppidi for helpful discussions and critical reading of the manuscript. M.A.M. was supported by 5F30AI131496-02 from the National Institute of Allergy and Infectious Diseases, J.G.C. is a Howard Hughes Medical Institute Investigator. J.H.F. is a Wellcome Trust Sir Henry Wellcome Fellow (107375/Z/15/Z), and M.L.D is a Wellcome Trust Principal Research Fellow (100262Z/12/Z). A.M. holds a Career Award for Medical Scientists from the Burroughs Wellcome Fund, is an investigator at the Chan Zuckerberg Biohub, is a Parker Institute for Cancer Immunotherapy investigator, and has received funding from the Innovative Genomics Institute. This work was supported in part by NIH grant R01 AI045073 (J.G.C), European Research Council Advanced Grant ERC-2014-AdG_670930 (M.L.D), and NIH grant P01 DK46763 (M.K).

AUTHOR CONTRIBUTIONS

M.A.M. and J.G.C. conceptualized the project, designed the experiments, interpreted the results, and wrote the manuscript. M.A.M performed the experiments. J.H.F., V.M., and M.L.D. designed and performed the lipid bilayer experiments, interpreted the results, and helped to prepare the manuscript. M.Y.C. performed CD40L induction studies, interpreted data, and helped to prepare the manuscript. Y.X. performed molecular biology experiments. J.A. genotyped mice. Z.L. and A.M. generated the BTLA Y3 mutant mouse line. T.O., J.-W.S., C.F.W., and M.K. provided gene-targeted mouse lines and input on the manuscript.

DECLARATION OF INTERESTS

A.M. is a co-founder of Arsenal Biosciences, Spotlight Therapeutics, and Sonoma Biotherapeutics. A.M. is on the scientific advisory board of PACT Pharma and was a former advisor for Juno Therapeutics. The Marson laboratory has received sponsored research support from Juno Therapeutics, Epinomics, and Sanofi and a gift from Gilead. J.G.C. is on the scientific advisory board of ALX Oncology and is a consultant for Corvus Pharmaceuticals.

Received: March 7, 2019
Revised: April 26, 2019
Accepted: May 29, 2019
Published: June 13, 2019

REFERENCES

Allen, C.D.C., Okada, T., Tang, H.L., and Cyster, J.G. (2007). Imaging of germinal center selection events during affinity maturation. Science *315*, 528–531.

Bannard, O., and Cyster, J.G. (2017). Germinal centers: programmed for affinity maturation and antibody diversification. Curr. Opin. Immunol. *45*, 21–30.

Bannard, O., McGowan, S.J., Ersching, J., Ishido, S., Victora, G.D., Shin, J.-S., and Cyster, J.G. (2016). Ubiquitin-mediated fluctuations in MHC class II facilitate efficient germinal center B cell responses. J. Exp. Med. *213*, 993–1009.

Boice, M., Salloum, D., Mourcin, F., Sanghvi, V., Amin, R., Oricchio, E., Jiang, M., Mottok, A., Denis-Lagache, N., Ciriello, G., et al. (2016). Loss of the HVEM tumor suppressor in lymphoma and restoration by modified CAR-T cells. Cell *167*, 405–418.e13.

Boisvert, J., Edmondson, S., and Krummel, M.F. (2004). Immunological synapse formation licenses CD40-CD40L accumulations at T-APC contact sites. J. Immunol. *173*, 3647–3652.

Bolotin, D.A., Poslavsky, S., Mitrophanov, I., Shugay, M., Mamedov, I.Z., Putintseva, E.V., and Chudakov, D.M. (2015). MiXCR: software for comprehensive adaptive immunity profiling. Nat. Methods *12*, 380–381.

Calado, D.P., Sasaki, Y., Godinho, S.A., Pellerin, A., Köchert, K., Sleckman, B.P., de Alborán, I.M., Janz, M., Rodig, S., and Rajewsky, K. (2012). The cell-cycle regulator c-Myc is essential for the formation and maintenance of germinal centers. Nat. Immunol. *13*, 1092–1100.

Casamayor-Palleja, M., Khan, M., and MacLennan, I.C. (1995). A subset of CD4+ memory T cells contains preformed CD40 ligand that is rapidly but transiently expressed on their surface after activation through the T cell receptor complex. J. Exp. Med. *181*, 1293–1301.

Chemnitz, J.M., Parry, R.V., Nichols, K.E., June, C.H., and Riley, J.L. (2004). SHP-1 and SHP-2 associate with immunoreceptor tyrosine-based switch motif of programmed death 1 upon primary human T cell stimulation, but only receptor ligation prevents T cell activation. J. Immunol. *173*, 945–954.

Chen, S., Lee, B., Lee, A.Y.-F., Modzelewski, A.J., and He, L. (2016). Highly efficient mouse genome editing by CRISPR ribonucleoprotein electroporation of zygotes. J. Biol. Chem. *291*, 14457–14467.

Cheung, T.C., Oborne, L.M., Steinberg, M.W., Macauley, M.G., Fukuyama, S., Sanjo, H., D'Souza, C., Norris, P.S., Pfeffer, K., Murphy, K.M., et al. (2009a). T cell intrinsic heterodimeric complexes between HVEM and BTLA determine receptivity to the surrounding microenvironment. J. Immunol. *183*, 7286–7296.

Cheung, T.C., Steinberg, M.W., Oborne, L.M., Macauley, M.G., Fukuyama, S., Sanjo, H., D'Souza, C., Norris, P.S., Pfeffer, K., Murphy, K.M., et al. (2009b). Unconventional ligand activation of herpesvirus entry mediator signals cell survival. Proc. Natl. Acad. Sci. USA *106*, 6244–6249.

Cheung, K.-J.J., Johnson, N.A., Affleck, J.G., Severson, T., Steidl, C., Ben-Neriah, S., Schein, J., Morin, R.D., Moore, R., Shah, S.P., et al. (2010). Acquired TNFRSF14 mutations in follicular lymphoma are associated with worse prognosis. Cancer Res. *70*, 9166–9174.

Choudhuri, K., Llodrá, J., Roth, E.W., Tsai, J., Gordo, S., Wucherpfennig, K.W., Kam, L.C., Stokes, D.L., and Dustin, M.L. (2014). Polarized release of T-cell-receptor-enriched microvesicles at the immunological synapse. Nature *507*, 118–123.

Chtanova, T., Tangye, S.G., Newton, R., Frank, N., Hodge, M.R., Rolph, M.S., and Mackay, C.R. (2004). T follicular helper cells express a distinctive transcriptional profile, reflecting their role as non-Th1/Th2 effector cells that provide help for B cells. J. Immunol. *173*, 68–78.

Cobaleda, C., Jochum, W., and Busslinger, M. (2007). Conversion of mature B cells into T cells by dedifferentiation to uncommitted progenitors. Nature *449*, 473–477.

de Jong, D., and Fest, T. (2011). The microenvironment in follicular lymphoma. Best Practice & Research Clinical Haematology *24*, 135–146.

Dominguez-Sola, D., Victora, G.D., Ying, C.Y., Phan, R.T., Saito, M., Nussenzweig, M.C., and Dalla-Favera, R. (2012). The proto-oncogene MYC is required for selection in the germinal center and cyclic reentry. Nat. Immunol. *13*, 1083–1091.

Dustin, M.L., Starr, T., Varma, R., and Thomas, V.K. (2007). Supported planar bilayers for study of the immunological synapse. Curr. Protoc. Immunol. *Chapter 18*, 13.

Ersching, J., Efeyan, A., Mesin, L., Jacobsen, J.T., Pasqual, G., Grabiner, B.C., Dominguez-Sola, D., Sabatini, D.M., and Victora, G.D. (2017). Germinal center selection and affinity maturation require dynamic regulation of mTORC1 kinase. Immunity *46*, 1045–1058.e6.

Gardell, J.L., and Parker, D.C. (2017). CD40L is transferred to antigen-presenting B cells during delivery of T-cell help. Eur. J. Immunol. *47*, 41–50.

Gavrieli, M., Watanabe, N., Loftin, S.K., Murphy, T.L., and Murphy, K.M. (2003). Characterization of phosphotyrosine binding motifs in the cytoplasmic domain of B and T lymphocyte attenuator required for association with protein tyrosine phosphatases SHP-1 and SHP-2. Biochem. Biophys. Res. Commun. *312*, 1236–1243.

Gitlin, A.D., Shulman, Z., and Nussenzweig, M.C. (2014). Clonal selection in the germinal centre by regulated proliferation and hypermutation. Nature *509*, 637–640.

Hsu, H., Solovyev, I., Colombero, A., Elliott, R., Kelley, M., and Boyle, W.J. (1997). ATAR, a novel tumor necrosis factor receptor family member, signals through TRAF2 and TRAF5. J. Biol. Chem. *272*, 13471–13474.

Huet, S., Sujobert, P., and Salles, G. (2018). From genetics to the clinic: a translational perspective on follicular lymphoma. Nat. Rev. Cancer *18*, 224–239.

Hui, E., Cheung, J., Zhu, J., Su, X., Taylor, M.J., Wallweber, H.A., Sasmal, D.K., Huang, J., Kim, J.M., Mellman, I., and Vale, R.D. (2017). T cell costimulatory receptor CD28 is a primary target for PD-1-mediated inhibition. Science *355*, 1428–1433.

Iezzi, G., Sonderegger, I., Ampenberger, F., Schmitz, N., Marsland, B.J., and Kopf, M. (2009). CD40-CD40L cross-talk integrates strong antigenic signals and microbial stimuli to induce development of IL-17-producing CD4+ T cells. Proc. Natl. Acad. Sci. USA *106*, 876–881.

Kashiwakuma, D., Suto, A., Hiramatsu, Y., Ikeda, K., Takatori, H., Suzuki, K., Kagami, S., Hirose, K., Watanabe, N., Iwamoto, I., and Nakajima, H. (2010). B and T lymphocyte attenuator suppresses IL-21 production from follicular Th cells and subsequent humoral immune responses. J. Immunol. *185*, 2730–2736.

Koguchi, Y., Thauland, T.J., Slifka, M.K., and Parker, D.C. (2007). Preformed CD40 ligand exists in secretory lysosomes in effector and memory CD4+ T cells and is quickly expressed on the cell surface in an antigen-specific manner. Blood *110*, 2520–2527.

Koguchi, Y., Buenafe, A.C., Thauland, T.J., Gardell, J.L., Bivins-Smith, E.R., Jacoby, D.B., Slifka, M.K., and Parker, D.C. (2012). Preformed CD40L is stored in Th1, Th2, Th17, and T follicular helper cells as well as CD4+ 8- thymocytes and invariant NKT cells but not in Treg cells. PLoS ONE *7*, e31296.

Kuleshov, M.V., Jones, M.R., Rouillard, A.D., Fernandez, N.F., Duan, Q., Wang, Z., Koplev, S., Jenkins, S.L., Jagodnik, K.M., Lachmann, A., et al. (2016). Enrichr: a comprehensive gene set enrichment analysis web server 2016 update. Nucleic Acids Res. *44* (W1), W90–W97.

Lackraj, T., Goswami, R., and Kridel, R. (2018). Pathogenesis of follicular lymphoma. Best Pract. Res. Clin. Haematol. *31*, 2–14.

Launay, E., Pangault, C., Bertrand, P., Jardin, F., Lamy, T., Tilly, H., Tarte, K., Bastard, C., and Fest, T. (2012). High rate of TNFRSF14 gene alterations related to 1p36 region in de novo follicular lymphoma and impact on prognosis. Leukemia *26*, 559–562.

Lavoie, T.B., Drohan, W.N., and Smith-Gill, S.J. (1992). Experimental analysis by site-directed mutagenesis of somatic mutation effects on affinity and fine specificity in antibodies specific for lysozyme. J. Immunol. *148*, 503–513.

Liu, D., Xu, H., Shih, C., Wan, Z., Ma, X., Ma, W., Luo, D., and Qi, H. (2015). T-B-cell entanglement and ICOSL-driven feed-forward regulation of germinal centre reaction. Nature *517*, 214–218.

Luo, W., Weisel, F., and Shlomchik, M.J. (2018). B cell receptor and CD40 signaling are rewired for synergistic induction of the c-Myc transcription factor in germinal center B cells. Immunity *48*, 313–326.e5.

Manso, B.A., Wenzl, K., Asmann, Y.W., Maurer, M.J., Manske, M., Yang, Z.-Z., Slager, S.L., Nowakowski, G.S., Ansell, S.M., Witzig, T.E., et al. (2017). Whole-exome analysis reveals novel somatic genomic alterations associated with cell of origin in diffuse large B-cell lymphoma. Blood Cancer J. *7*, e553.

Mayya, V., Judokusumo, E., Abu Shah, E., Peel, C.G., Neiswanger, W., Depoil, D., Blair, D.A., Wiggins, C.H., Kam, L.C., and Dustin, M.L. (2018). Durable interactions of T cells with T cell receptor stimuli in the absence of a stable immunological synapse. Cell Rep. *22*, 340–349.

Mesin, L., Ersching, J., and Victora, G.D. (2016). Germinal center B cell dynamics. Immunity *45*, 471–482.

Muppidi, J.R., Schmitz, R., Green, J.A., Xiao, W., Larsen, A.B., Braun, S.E., An, J., Xu, Y., Rosenwald, A., Ott, G., et al. (2014). Loss of signalling via Gα13 in germinal centre B-cell-derived lymphoma. Nature *516*, 254–258.

Murphy, K.M., Nelson, C.A., and Sedý, J.R. (2006). Balancing co-stimulation and inhibition with BTLA and HVEM. Nat. Rev. Immunol. *6*, 671–681.

Nurieva, R.I., Chung, Y., Martinez, G.J., Yang, X.O., Tanaka, S., Matskevitch, T.D., Wang, Y.-H., and Dong, C. (2009). Bcl6 mediates the development of T follicular helper cells. Science *325*, 1001–1005.

Ogilvy, S., Metcalf, D., Print, C.G., Bath, M.L., Harris, A.W., and Adams, J.M. (1999). Constitutive Bcl-2 expression throughout the hematopoietic compartment affects multiple lineages and enhances progenitor cell survival. Proc. Natl. Acad. Sci. USA *96*, 14943–14948.

Owada, T., Watanabe, N., Oki, M., Oya, Y., Saito, Y., Saito, T., Iwamoto, I., Murphy, T.L., Murphy, K.M., and Nakajima, H. (2010). Activation-induced accumulation of B and T lymphocyte attenuator at the immunological synapse in CD4+ T cells. J. Leukoc. Biol. *87*, 425–432.

Oya, Y., Watanabe, N., Owada, T., Oki, M., Hirose, K., Suto, A., Kagami, S., Nakajima, H., Kishimoto, T., Iwamoto, I., et al. (2008). Development of autoimmune hepatitis-like disease and production of autoantibodies to nuclear antigens in mice lacking B and T lymphocyte attenuator. Arthritis Rheum. *58*, 2498–2510.

Papa, I., Saliba, D., Ponzoni, M., Bustamante, S., Canete, P.F., Gonzalez-Figueroa, P., McNamara, H.A., Valvo, S., Grimbaldeston, M., Sweet, R.A., et al. (2017). T_{FH}-derived dopamine accelerates productive synapses in germinal centres. Nature *547*, 318–323.

Paus, D., Phan, T.G., Chan, T.D., Gardam, S., Basten, A., and Brink, R. (2006). Antigen recognition strength regulates the choice between extrafollicular plasma cell and germinal center B cell differentiation. J. Exp. Med. *203*, 1081–1091.

Ritthipichai, K., Haymaker, C.L., Martinez, M., Aschenbrenner, A., Yi, X., Zhang, M., Kale, C., Vence, L.M., Roszik, J., Hailemichael, Y., et al. (2017). Multifaceted role of BTLA in the control of $CD8^+$ T-cell fate after antigen encounter. Clin. Cancer Res. *23*, 6151–6164.

Rota, G., Niogret, C., Dang, A.T., Barros, C.R., Fonta, N.P., Alfei, F., Morgado, L., Zehn, D., Birchmeier, W., Vivier, E., and Guarda, G. (2018). Shp-2 is dispensable for establishing T cell exhaustion and for PD-1 signaling in vivo. Cell Rep. *23*, 39–49.

Ruedl, C., Bachmann, M.F., and Kopf, M. (2000). The antigen dose determines T helper subset development by regulation of CD40 ligand. Eur. J. Immunol. *30*, 2056–2064.

Schmitz, R., Wright, G.W., Huang, D.W., Johnson, C.A., Phelan, J.D., Wang, J.Q., Roulland, S., Kasbekar, M., Young, R.M., Shaffer, A.L., et al. (2018). Genetics and pathogenesis of diffuse large B-cell lymphoma. N. Engl. J. Med. *378*, 1396–1407.

Schwickert, T.A., Victora, G.D., Fooksman, D.R., Kamphorst, A.O., Mugnier, M.R., Gitlin, A.D., Dustin, M.L., and Nussenzweig, M.C. (2011). A dynamic T cell-limited checkpoint regulates affinity-dependent B cell entry into the germinal center. J. Exp. Med. *208*, 1243–1252.

Sedy, J.R., Gavrieli, M., Potter, K.G., Hurchla, M.A., Lindsley, R.C., Hildner, K., Scheu, S., Pfeffer, K., Ware, C.F., Murphy, T.L., and Murphy, K.M. (2005). B and T lymphocyte attenuator regulates T cell activation through interaction with herpesvirus entry mediator. Nat. Immunol. *6*, 90–98.

Seo, G.-Y., Shui, J.-W., Takahashi, D., Song, C., Wang, Q., Kim, K., Mikulski, Z., Chandra, S., Giles, D.A., Zahner, S., et al. (2018). LIGHT-HVEM signaling in innate lymphoid cell subsets protects against enteric bacterial infection. Cell Host Microbe *24*, 249–260.e4.

Shi, J., Hou, S., Fang, Q., Liu, X., Liu, X., and Qi, H. (2018). PD-1 controls follicular T helper cell positioning and function. Immunity *49*, 264–274.e4.

Shinnakasu, R., Inoue, T., Kometani, K., Moriyama, S., Adachi, Y., Nakayama, M., Takahashi, Y., Fukuyama, H., Okada, T., and Kurosaki, T. (2016). Regulated selection of germinal-center cells into the memory B cell compartment. Nat. Immunol. *17*, 861–869.

Shui, J.-W., Larange, A., Kim, G., Vela, J.L., Zahner, S., Cheroutre, H., and Kronenberg, M. (2012). HVEM signalling at mucosal barriers provides host defence against pathogenic bacteria. Nature *488*, 222–225.

Steinberg, M.W., Cheung, T.C., and Ware, C.F. (2011). The signaling networks of the herpesvirus entry mediator (TNFRSF14) in immune regulation. Immunol. Rev. *244*, 169–187.

Subramanian, A., Tamayo, P., Mootha, V.K., Mukherjee, S., Ebert, B.L., Gillette, M.A., Paulovich, A., Pomeroy, S.L., Golub, T.R., Lander, E.S., et al. (2005). Gene set enrichment analysis: a knowledge-based approach for interpreting genome-wide expression profiles. Proc. Nat. Acad. Sci. *102*, 15545–15550.

Vendel, A.C., Calemine-Fenaux, J., Izrael-Tomasevic, A., Chauhan, V., Arnott, D., and Eaton, D.L. (2009). B and T lymphocyte attenuator regulates B cell receptor signaling by targeting Syk and BLNK. J. Immunol. *182*, 1509–1517.

Verdière, L., Mourcin, F., and Tarte, K. (2018). Microenvironment signaling driving lymphomagenesis. Curr. Opin. Hematol. *25*, 335–345.

Wakamatsu, E., Mathis, D., and Benoist, C. (2013). Convergent and divergent effects of costimulatory molecules in conventional and regulatory CD4+ T cells. Proc. Natl. Acad. Sci. USA *110*, 1023–1028.

Wang, Y., Subudhi, S.K., Anders, R.A., Lo, J., Sun, Y., Blink, S., Wang, Y., Wang, J., Liu, X., Mink, K., et al. (2005). The role of herpesvirus entry mediator as a negative regulator of T cell-mediated responses. J. Clin. Invest. *115*, 711–717.

Ward-Kavanagh, L.K., Lin, W.W., Šedý, J.R., and Ware, C.F. (2016). The TNF receptor superfamily in co-stimulating and co-inhibitory responses. Immunity *44*, 1005–1019.

Watanabe, N., Gavrieli, M., Sedy, J.R., Yang, J., Fallarino, F., Loftin, S.K., Hurchla, M.A., Zimmerman, N., Sim, J., Zang, X., et al. (2003). BTLA is a lymphocyte inhibitory receptor with similarities to CTLA-4 and PD-1. Nat. Immunol. *4*, 670–679.

Wu, T.-H., Zhen, Y., Zeng, C., Yi, H.-F., and Zhao, Y. (2007). B and T lymphocyte attenuator interacts with CD3ζ and inhibits tyrosine phosphorylation of TCRζ complex during T-cell activation. Immunol. Cell Biol. *85*, 590–595.

Xu, H., Li, X., Liu, D., Li, J., Zhang, X., Chen, X., Hou, S., Peng, L., Xu, C., Liu, W., et al. (2013). Follicular T-helper cell recruitment governed by bystander B cells and ICOS-driven motility. Nature *496*, 523–527.

Yang, Z., Sullivan, B.M., and Allen, C.D.C. (2012). Fluorescent in vivo detection reveals that IgE^+ B cells are restrained by an intrinsic cell fate predisposition. Immunity *36*, 857–872.

Yeh, C.-H., Nojima, T., Kuraoka, M., and Kelsoe, G. (2018). Germinal center entry not selection of B cells is controlled by peptide-MHCII complex density. Nat. Commun. *9*, 928.

Yi, T., and Cyster, J.G. (2013). EBI2-mediated bridging channel positioning supports splenic dendritic cell homeostasis and particulate antigen capture. eLife *2*, e00757.

Yokosuka, T., Takamatsu, M., Kobayashi-Imanishi, W., Hashimoto-Tane, A., Azuma, M., and Saito, T. (2012). Programmed cell death 1 forms negative costimulatory microclusters that directly inhibit T cell receptor signaling by recruiting phosphatase SHP2. J. Exp. Med. *209*, 1201–1217.

Yu, D., Cozma, D., Park, A., and Thomas-Tikhonenko, A. (2005). Functional validation of genes implicated in lymphomagenesis: an in vivo selection assay using a Myc-induced B-cell tumor. Ann. N Y Acad. Sci. *1059*, 145–159.

Zaretsky, I., Atrakchi, O., Mazor, R.D., Stoler-Barak, L., Biram, A., Feigelson, S.W., Gitlin, A.D., Engelhardt, B., and Shulman, Z. (2017). ICAMs support B cell interactions with T follicular helper cells and promote clonal selection. J. Exp. Med. *214*, 3435–3448.

Zhao, Y., Zheng, Z., Cohen, C.J., Gattinoni, L., Palmer, D.C., Restifo, N.P., Rosenberg, S.A., and Morgan, R.A. (2006). High-efficiency transfection of primary human and mouse T lymphocytes using RNA electroporation. Mol. Ther. *13*, 151–159.

STAR★METHODS

KEY RESOURCES TABLE

REAGENT or RESOURCE	SOURCE	IDENTIFIER
Antibodies		
B220 APC-CY7 (clone RA3-6B2)	BD Bioscience	#552094; RRID: AB_394335
B220 BV785 (clone RA3-6B2)	BioLegend	#103246
IgD FITC (clone 11-26c.2a)	BioLegend	#405704; RRID: AB_394859
GL-7 Pacific Blue (clone GL7)	BioLegend	#144614
FAS PE-CY7 (clone Jo2)	BD Bioscience	#557653
CD38 AlexaFluor647 (clone 90)	BioLegend	#102718
CD45.1 PERCP-CY5.5, APC (clone A20)	Tonbo	#65-0453-U100
CD45.2 BV605 (clone 104)	BioLegend	#109841
CD45.2 APC-CY7 (clone 104)	BioLegend	#109824; RRID: AB_1727492
HVEM PE (clone LH1)	eBioscience	#12-5962-80; RRID: AB_953628
BTLA AlexaFluor 647 (8F4)	BioLegend	#134808
Ephrin-B1 Biotin (polyclonal, R&D)	R&D	#BAF473
CXCR4 Biotin (clone 2B11/CXCR4)	BD Bioscience	#551968
CD86 AlexaFluor647 (clone GL-1)	BioLegend	#105020
IgG1 FITC (clone RMG1-1)	BioLegend	#406606
CD138 BV421 (clone 281-2)	BioLegend	#142508
CD73 PERCP-CY5.5 (clone TY/11.8)	BioLegend	#127214
CD4 PE-CY7 (clone GK1.5)	BD Bioscience	#552775
TCRβ Pacific Blue (clone H57-597)	BioLegend	#109226
CXCR5 BV605 (clone L138D7)	BioLegend	#145513
PD-1 PE (clone 29F.1A12)	BioLegend	#135206
PD-1 FITC (clone 29F.1A12)	BioLegend	#135214
CD40L PE (clone MR1)	BioLegend	#106506; RRID: AB_313270
Vα2 PERCP-CY5.5 (clone B20.1)	BD Bioscience	#560529
Fixable viability dye eFluor780 (eBioscience)	eBioscience	#65-0865-18
NP-47-PE (Biosearch technologies)	Biosearch Technologies	#N-5070-1
Active caspase-3 (clone C92-605)	BD Bioscience	#559565
BTLA AlexaFluor 647 (clone MIH26)	BioLegend	#344520
CXCR5 AlexaFluor 488 (clone RF8B2)	BD Bioscience	#558112
CXCR5 PE/Cy7 (clone J252D4)	BioLegend	#356923
PD1 AlexaFluor 488 (clone EH12.2H7)	BioLegend	#329935
PD1 AlexaFluor 647 (clone EH12.2H7)	BioLegend	#329910
CD19 PE/Cy7 (clone SJ25C1)	BD Bioscience	#560911
CD38 AlexaFluor 488 (clone HIT2)	BioLegend	#303511
CD27 BrilliantViolet421 (clone M-T271)	BioLegend	#356417
CD20 PE (clone 2H7)	BioLegend	#302305
HVEM AlexaFluor 647 (clone 94801)	BD Bioscience	#564411
SHP1 (clone C-19)	Santa Cruz Biotechnology	#sc-287
SHP2 (clone C-18)	Santa Cruz Biotechnology	#sc-280; RRID: AB_632401
pT538 PKCθ (polyclonal)	Cell Signaling Technology	#9377
pY493 ZAP70 (polyclonal)	Cell Signaling Technology	#2704
Goat anti-rabbit F(ab')2 AlexaFluor 568 (polyclonal)	Thermo Fisher Scientific	#A-21069
Anti-CD3 monobiotinylated (clone OKT3)	eBioscience	#13-0037-82

(*Continued on next page*)

Continued

REAGENT or RESOURCE	SOURCE	IDENTIFIER
Bacterial and Virus Strains		
5-alpha F'Iq Competent *E. coli* (High Efficiency)	New England Bioscience	#C2992H
Biological Samples		
Healthy adult blood – leukocyte cones	UK NHS Blood and Transplant	https://www.nhsbt.nhs.uk/
Adult tonsillectomy tissue – whole tonsils	UK NHS Blood and Transplant	https://www.nhsbt.nhs.uk/
Chemicals, Peptides, and Recombinant Proteins		
4-Hydroxy-3-nitrophenylacetyl (NP)-20-29-Chicken gamma globulin (CGG)	Biosearch Technologies	#N-5055C-5
Alhydrogel aluminum hydroxide (alum) gel 6.5 mg/mL	Accurate Chemical & Scientific Corporation	#A1090S
OVA peptide (323-339)	GenScript	#RP10610
Human IL-2	Peprotech	#200-02
1,2-dioleoyl-sn-glycero-3-phosphocholine	Avanti Polar Lipids	#860377P
1,2-dioleoyl-sn-glycero-3-[(N-(5-amino-1-carboxypentyl) iminodiacetic acid) succinyl]-Ni	Avanti Polar Lipids	#790404P
Human HVEM-Fc-His	Simon Davis Laboratory	N/A
Human ICAM1-His	Michael Dustin Laboratory	N/A
Human CD40-His	Michael Dustin Laboratory	N/A
Human CD80-His	Michael Dustin Laboratory	N/A
Anti-Human CD3 FaB-His (clone UCHT1)	Michael Dustin Laboratory	N/A
SNAP-Cell 647-SiR ligand	New England Biolabs	#S9102
Human CD58-His	Michael Dustin Laboratory	N/A
Human PDL1-His	Michael Dustin Laboratory	N/A
Critical Commercial Assays		
Click-iT Plus EdU Alexa Fluor 647 Flow Cytometry Assay Kit 100 Tests	Invitrogen	#C10635
RosetteSep Human CD4+ T Cell Enrichment Cocktail	StemCell Technologies	#15062
Anti-Human CD3/CD28 Dynabeads	GIBCO	#111.32D
mMESSAGE mMACHINE T7 ULTRA Transcription Kit	Thermo Fisher Scientific	#AM1345
EasySep CD4+ Isolation Kit	StemCell Technologies	#17952
Deposited Data		
RNA sequencing data	This paper	GEO: GSE130095
Experimental Models: Cell Lines		
Platinum-E (Plat-E) Retroviral Packaging Cell Line	Gift from S. Schwab	N/A
Experimental Models: Organisms/Strains		
Mouse: B6-CD45.1 Strain ID: B6.SJL-*Ptprc*^{a}*Pepc*^{b}/BoyCrCrl	NCI at Charles River	Stock No: 564
Mouse: *Hvem* flox Strain ID: B6;SJL-*Tnfrsf14*$^{tm1.1Kro}$/J	Jackson Laboratories	Stock No: 030862
Mouse: *Hvem*$^{-/-}$ derived from *Hvem* flox	Seo et al., 2018	N/A
Mouse: *Hvem14*$^{-/-}$	Wang et al., 2005	N/A
Mouse: *Btla* flox	This paper	N/A
Mouse: *Btla*$^{-/-}$ Strain ID: B6.129-*Btla*^{tm1Kmm}/J	Jackson Laboratories	Stock No: 008336
Mouse: *Ptpn6* flox Strain ID: B6.129P2-*Ptpn6*^{tm1Rsky}/J	Jackson Laboratories	Stock No.: 008336
Mouse: *Ptpn11* flox Strain ID: *Ptpn11*$^{tm1.1Wbm}$/J	Jackson Laboratories	Stock No.: 025758
Mouse: *Bcl6* flox Strain ID: B6.129S(FVB)-*Bcl6*$^{tm1.1Dent}$/J	Jackson Laboratories	Stock No.: 023727
Mouse: *C*γ*1*-Cre Strain ID: B6.129P2(Cg)-*Ighg1*$^{tm1(cre)Cgn}$/J	Jackson Laboratories	Stock No.: 010611
Mouse: *S1pr2*-ERT2Cre tdTomato Flox	Shinnakasu et al., 2016	N/A
Mouse: *Mb1*-Cre Strain ID: B6.C(Cg)-*Cd79a*$^{tm1(cre)Reth}$/EhobJ	Jackson Laboratories	Stock No.: 020505
Mouse: *Cd4*-Cre Strain ID: B6.Cg-Tg(Cd4-cre)1Cwi/BfluJ	Jackson Laboratories	Stock No.: 022071
Mouse: Hy10 (VDJ9/K5)	Allen et al., 2007	N/A

(*Continued on next page*)

Continued

REAGENT or RESOURCE	SOURCE	IDENTIFIER
Mouse: OT-II Strain ID: Tg(TcraTcrb)426-6Cbn	MGI 4836972	N/A
Mouse: *Rag2*-deficient Strain ID: B6(Cg)-*Rag2*$^{tm1.1Cgn}$/J	Jackson Laboratories	008449
Mouse: Eμ-*BCL2*-tg Strain ID: B6.Cg-Tg(BCL2)22Wehi/J	Jackson Laboratories	002319
Mouse: *Tcrb*$^{-/-}$ Strain ID: B6.129P2-*Tcrb*^{tm1Mom}/J	Jackson Laboratories	002118
Mouse: *Btla*-Y3	This paper	This paper
Oligonucleotides		
Hvem (*Tnfrsf14*) qPCR primers: GGAGCTGGGATAGCT GGATTC (forward), TCTCCTGTTGTTCCTGGAAAGG (reverse)	N/A	N/A
Il-21 qPCR primers: GCTCCACAAGATGTAAAGGG (forward), TTATTGTTTCCAGGGTTTGA (reverse)	N/A	N/A
Vh186.2 PCR1 primers: CATGGGATGGAGCTGTATCATGC (forward), CTCACAAGAGTCCGATAGACCCTG (reverse); PCR2 nested: GGTGACAATGACATCCACTTTGC (forward), GACTGTGAGAGTGGTGCCTTG (reverse)	N/A	N/A
Vh186.2 186-EF: GTATCATGCTCTTCTTGGCAGC; 186-IF: ACAGTAGCAGGCTTGAGGTCTG; 186-IR: CCCAATGACC CTTTCTGACTC; 186-ER: TGAGGATGTCTGTCTGCGTCA	N/A	N/A
Recombinant DNA		
MSCV-IRES-Thy1.1	Addgene	Plasmid ID: 17442
Software and Algorithms		
Prism 9	GraphPad Software	http://www.graphpad.com/ scientific-software/ prism/
GSEA v3.0	Subramanian et al., 2005	http://software.broadinstitute. org/gsea/index.jsp
Flowjo v9	FlowJo	https://www.flowjo.com/
Adobe Illustrator 2019	Adobe Systems	N/A
ImageJ	NIH	https://imagej.nih.gov/ij/

LEAD CONTACT AND MATERIALS AVAILABILITY

Further information and requests for resources and reagents should be directed to and will be fulfilled by the Lead Contact, Jason G. Cyster (jason.cyster@ucsf.edu). The mouse lines obtained from other laboratories are described below and may require a Material Transfer Agreement (MTA) with the providing scientists. BTLAY3 mice generated in this study are available from our laboratory, also with an MTA.

EXPERIMENTAL MODEL AND SUBJECT DETAILS

Mice

Adult C57BL6 CD45.1 mice at least 7 weeks of age were from the National Cancer Institute. *Hvem* flox animals (JAX stock 030862) and *Hvem*-deficient animals (Seo et al., 2018) were used in most studies. In one experiment mixed chimeras were generated from a separate *Hvem*-deficient line (Wang et al., 2005). *Btla* flox animals have loxp sites flanking exons 4 and 5 of *Btla* gene (J.-W.S. and M.K., unpublished data). Full *Btla* deficient mice were from JAX stock 006353 (Watanabe et al., 2003). Other lines included *Ptpn6* flox (JAX stock 008336), *Ptpn11* flox (JAX stock 025758), *Bcl6* flox (JAX 023727), *C*γ*1*-Cre (JAX stock 010611), *S1pr2*-ERT2Cre tdTomato Flox (Shinnakasu et al., 2016), *Mb1*-Cre (JAX stock 020505), *Cd4*-Cre (JAX stock 022071), Hy10 (Allen et al., 2007), OT-II (MGI 4836972), and *Rag2*$^{-/-}$ (JAX stock 008449), *Tcrb*$^{-/-}$ (JAX stock 002118), Eμ-BCL2-tg (JAX stock 002319), and *Btla*-y3/y3 animals were generated and maintained in our laboratory. In most experiments, littermates were used as controls and experimental animals were co-caged in groups of 2–6 whenever possible. Male and female mice were used as both donors and recipients, except for OT-II animals, which required male donors. Animals were housed in specific pathogen free environment in the Laboratory Animal Research Center at UCSF and all experiments conformed to ethical principles and guidelines approved by the UCSF Institutional Animal Care and Use Committee.

METHOD DETAILS

Generation of BTLAY3 Mice

Btla-y3/y3 animals were generated using crRNA1:TCATAAATTCCAGTTCCTGA targeting guide. The protocol followed Chen et al. (Chen et al., 2016) with the main exception that the standard square curve electroporation was performed twice with an interval of 3 s. RNP assembly followed standard protocol: 160 μM tracrRNA + 160 μM crRNA (Dharmacon), equal volume mix well 37°C, 30 min (80 μM sgRNA); 80 μM sgRNA + 40 μM Cas9 Protein equal volume mix well 37°C 10 min (20 μM RNPs); leave on ice, each electroporation 10 μL of RNPs mix with 10 μL Opti-MEM with C57B6/J embryos. Standard electroporation: two pulses 30V for 3 ms interval 100 ms. Embryos were transferred into pseudo-pregnant females.

Bone Marrow Chimeras

WT CD45.1, or *Rag2*$^{-/-}$, or *Tcrb*$^{-/-}$ where indicated, were lethally irradiated with 1,100 rads gamma-irradiation (split dose separated by 3 h) and then i.v. injected with relevant BM cells. BM was harvested by flushing the tibia and femurs. For T cell depletion (when necessary), 250 μg of anti-CD4 GK1.5 (BioXcell) was injected i.v. at day −1 and day 0.

Retroviral Constructs and Transductions

Murine HVEM and Bcl2 retroviral constructs were made by inserting the mouse open reading frame into the MSCV2.2 retroviral vector followed by an internal ribosome entry site (IRES) and Thy1.1 as an expression marker. *Hvem* point mutations were introduced by quick-change PCR and *Hvem* truncation was introduced by PCR. Retrovirus was generated by transfecting PLAT-E packaging cell line with 10 μg plasmid DNA and 10 μg Lipofectamine 2000 (Fischer). For transduction of BM, WT, *Hvem*$^{-/-}$, or *Btla*$^{-/-}$ mice were injected i.v. with 3 mg 5-fluorouracil (Sigma). BM was collected after 4 days and cultured in DMEM containing 15% (vol/vol) FBS, antibiotics (penicillin (50 IU/mL) and streptomycin (50 μg/mL); Cellgro) and 10 mM HEPES, pH 7.2 (Cellgro), supplemented with IL-3, IL-6 and stem cell factor (at concentrations of 20, 50 and 100 ng/mL, respectively; Peprotech). Cells were 'spin-infected' twice at days 1 and 2 and were transferred into irradiated recipients on day 3.

Cell Isolation and Adoptive Transfer

Spleens were macerated and resulting cell suspensions were filtered through a 70 μm mesh into PBS supplemented with 2% FCS and 1mM EDTA. Cells were counted on a hemocytometer and frequency of HEL^{+} Hy10 B cell was determined by staining of HEL-AlexaFluor647 (made using Invitrogen Alexa Fluor 647 antibody labeling kit) positive B cells on the flow cytometer. Mixes of *Hvem*$^{-/-}$ or *Hvem*$^{+/+}$ Hy10 and WT Hy10 congenically mismatched B cells were made at ratios of 1:4-1:1 as indicated in the figures. In some experiments OT-II T cells were co-transferred at a ratio of 1:5 Hy10 B cell as indicated in the figures. Mixtures of 5×10^{4} - 2×10^{5} cells were transferred i.v. in a volume of 200 μL into the retro-orbital venous sinus. To visualize cell proliferation, cells were labeled with Cell Trace Violet (CTV) (Invitrogen).

Immunizations

Hosts were immunized with an intermediate affinity mutant 2x-HEL (Paus et al., 2006) (Gift of R. Brink) conjugated to SRBC (Colorado Serum Company) (Yi and Cyster, 2013) intraperitoneally (i.p.). If Hy10 B cells were co-transferred with OT-II, hosts were immunized with 50 μg of HEL-OVA or DEL-OVA (Yi and Cyster, 2013) mixed 1:1 with Sigma Adjuvant System (SAS, previously Sigma RIBI adjuvant) for a total volume of 200 μL. Animals were immunized with 100 μg NP-(20-29)-CGG (Biosearch technologies) mixed with 1:1 Alum (Alhydrogel) for a total of 200 μL volume. 2×10^{8} SRBC (Colorado Serum Company) were injected in a volume of 300 μL. Tamoxifen (Sigma) was dissolved in Corn Oil (Sigma) at 20 mg/mL and injected at 2 mg/20 g mouse i.p. TAM diet (Envigo) containing chow replaced normal chow when indicated.

Flow Cytometry

Cells were stained on ice, in 96 round bottom plates in PBS supplemented with 2% FBS and 1mM EDTA. The following antibodies were used: B220 APC-CY7 or BV785 (clone RA3-6B2), IgD FITC (clone 11-26c.2a), GL-7 Pacific Blue (clone GL7), FAS PE-CY7 (clone Jo2), CD38 AlexaFluor647 (clone 90), CD45.1 PERCP-CY5.5, APC (clone A20), CD45.2 BV605, APC-CY7 (clone 104), HVEM PE (clone LH1), BTLA AlexaFluor 647 (8F4), Ephrin-B1 Biotin (polyclonal, R&D), CXCR4 Biotin (clone 2B11/CXCR4), CD86 AlexaFluor647 (clone GL-1), IgG1 FITC (clone RMG1-1), CD138 BV421 (clone 281-2), CD73 PERCP-CY5.5 (clone TY/11.8), CD4 PE-CY7 (clone GK1.5), TCRβ Pacific Blue (clone H57-597), CXCR5 BV605 (clone L138D7), PD-1 PE or FITC (clone 29F.1A12), CD40L PE (clone MR1), Vα2 PERCP-CY5.5 (clone B20.1), Active caspase-3 (clone C92-605), Fixable viability dye eFluor780 (eBioscience). NP-47-PE (Biosearch technologies). For intracellular staining, cells were fixed and permeabilized using BD Cytofix/Cytoperm kit. EdU was detected with Click-IT Plus EdU kit (Invitrogen). Data were collected on a BD LSRII and analyzed on FlowJo Software.

qPCR and RNA Sequencing

Hvem primers F: GGAGCTGGGATAGCTGGATTC R: TCTCCTGTTGTTCCTGGAAAGG, *Il21* primers F: GCTCCACAAGATG TAAAGGG R: TTATTGTTTCCAGGGTTTGA. Cells were sorted using BD FACSAria II. 10^{4} cells or greater were sorted for qPCR and 5×10^{4} cells were sorted for RNA-sequencing. RNA sequencing was prepared using Ovation RNA-seq System V2 from Nugen,

KAPA Hyper prep labeling kit, NEXTflex DNA barcodes Adapter kit from Bioo Scientific. 100 bp paired end was run on HiSeq4000 at UCSF Institute for Human Genetics. Data were run through STAR alignment and Deseq2.

GSEA and Gene Ontology

GSEA analysis (v3.0) through the BROAD was performed using standard settings: 1000 number of permutations, collapsed data to gene symbols, permutation type phenotype, enrichment statistic weighted. Data for Rapamycin sensitive DEC205-WT GC B cell genes were accessed (Ersching et al., 2017) and significant genes, defined as padj < 0.01 with > 2-fold increased expression in untreated v. Rapa treated, were made into a manually curated gene list. Yu Myc targets UP was a premade GSEA gene list. Gene ontology was performed using Enrichr platform (Kuleshov et al., 2016).

NP V_H186.2 Mutation Analysis

For bulk GC sequencing: PCR reaction 1. PCR1 F primer: CATGGGATGGAGCTGTATCATGC R primer: CTCACAAGAGTCCGA TAGACCCTG PCR2 (nested) F primer: GGTGACAATGACATCCACTTTGC R primer: GACTGTGAGAGTGGTGCCTTG and the blunt end cloned into TOPO vector. Plates were sent to TacGen for plasmid prep and Sanger sequencing (Bannard et al., 2016). For single cell sequencing: NP^+ cells were sorted into lysis buffer and frozen in 96-well round bottom plates. Nested PCR with primers and sent for sequencing. 186-EF: GTATCATGCTCTTCTTGGCAGC 186-IF: ACAGTAGCAGGCTTGAGGTCTG 186-IR: CCCAAT GACCCTTTCTGACTC 186-ER: TGAGGATGTCTGTCTGCGTCA (Yang et al., 2012). Data were analyzed using IgBLAST and the frequency of W33L were counted in non-frame shifted IGHV1-72*01 sequences.

Tfh Cell CD40L Surface Mobilization

Tfh cells were generated by transferring OT-II into WT mice on day -1 and immunizing retro-orbitally with 2×10^8 SRBCs (Colorado Serum Company) conjugated to 10 μg (0.85 mg/mL) HEL-OVA and mixed with 150 μg (1 mg/mL) poly I:C (GE Healthcare) pre-heated for 10 min at 60°C in a total volume of 350 μL on day 0. On day 3, spleens were harvested and $CD4^+$ T cells were isolated through negative selection by depletion with anti-CD8a biotin, anti-CD11c biotin, and anti-CD19 biotin antibodies and EasySep Mouse Streptavidin RapidSpheres (Stemcell) in complete RPMI. For B cells, spleens were harvested from WT or $Hvem^{-/-}$ mice. Splenocytes were CTV labeled at 0.5 μM and pulsed with 10 μM, 2 μM, 0.4 μM, or 0.08 μM OVA 323-339 peptide (GenScript) antigen or no antigen for 2 h at 37°C. The splenocytes were all washed three times and 8×10^5 splenocytes were combined with 4×10^5 $CD4^+$ T cells in a 96-well round bottom plate. To facilitate detection of surface exposed CD40L, the cells were incubated in the presence of 1 μg/mL anti-CD40L PE antibody as previously described (Gardell and Parker, 2017; Koguchi et al., 2007). After 30 or 60 min incubation at 37°C, T cells were harvested, stained, and analyzed on the LSRII flow cytometer. Anti-CD11c biotin antibody and EasySep Mouse Streptavidin RapidSpheres (Stemcell) were used to deplete dendritic cells in two experiments with no differing results.

Assessment of BCR Clonality by PCR

Assessment of clonality by PCR of J558 heavy chain, and κ light chains from genomic DNA from 2×10^4 FACS sorted GC B cells from 8 week old *Btla*$^{f/f}$ *Cd4*^{Cre} and *Btla*$^{f/f}$ control chimeras (Cobaleda et al., 2007; Muppidi et al., 2014). Primers:

V_HJ558 CGAGCTCTCCARCACAGCCTWCATGCARCTCARC
J_H4 CGAGCTCTCCARCACAGCCTWCATGCARCTCARC
V_κ GGCTGCAGSTTCAGTGGCAGTGGRTCWGGRAC
J_κ5 GGCTGCAGSTTCAGTGGCAGTGGRTCWGGRAC

Human $CD4^+$ T Cell Isolation, Stimulation, And Transfection

Primary human $CD4^+$ T cells were isolated using the RosetteSep Human $CD4^+$ T Cell Enrichment Cocktail (StemCell Technologies) as per the manufacturer's instructions from leukocyte cones provided by UK National Health Service Blood and Transplant. Use of leukapheresis products at the University of Oxford was approved by the Non-Clinical Issue division of the National Health Service (REC 11/H11/7), and the use of human tonsil tissue was approved under the Oxford Radcliffe Biobank (ORB) research tissue bank ethics, reference 09/H0606/5+5. Isolated cells were cultured in RPMI-1640 supplemented with 10% FCS, 4 mM L-glutamine, 10 mM HEPES, 1% non-essential amino acid solution (GIBCO), and 1% penicillin-streptomycin solution (GIBCO) at 37°C, 5% CO_2 for between 24 and 72 h before stimulating. Cells were diluted to 1×10^6/mL in supplemented RPMI-1640 containing 50 U/mL recombinant IL-2 (PeproTech) and anti-human CD3/CD28 Dynabeads (GIBCO) at 1×10^6/mL. Cells were cultured for 3 days before the beads were removed by magnetic separation and the medium replaced with fresh supplemented RPMI-1640 + 50 U/mL IL-2. Cells were cultured for a further 4 days with medium replaced and cells diluted to 1×10^6/mL as required.

Human BTLA was cloned into pGEM-SnapTag vector. This vector was used to produce mRNA *in vitro* using the mMESSAGE mMACHINE T7 Transcription Kit (Thermo Fisher Scientific) as per the manufacturer's instructions. Transfection was performed 24 h before imaging. Cells were washed 3 times with OptiMEM (GIBCO) at room temperature and resuspended at 2.5×10^6 cells/100 μl. 2.5 – 7.5 μg mRNA encoding WT or mutant BTLA-SNAP-tag was added to 2.5×10^6 cells, which were gently mixed, transferred to a Gene Pulser cuvette (BioRad) and pulsed for 2 ms at 300 V in an ECM 830 Square Wave Electroporation System (BTX). Cells were then immediately transferred to supplemented RPMI-1640 at 1×10^6/mL and cultured for 24 h. The amount of

mRNA used was optimized for each T cell donor and mRNA preparation by performing multiple transfections with titrated mRNA amounts, then assessing total BTLA expression after 24 h by staining with AlexaFluor 647-conjugated anti-BTLA (BioLegend, 344520) and comparing to Quantum AlexaFluor 647 MESF calibration beads (Bangs Laboratories) by flow cytometry. Conditions giving ~10,000 transfected BTLA/cell were selected for imaging.

Human Tfh Cell Isolation

Human Tfh cells were isolated from tonsils provided by UK National Health Service Blood and Transplant. Tissue was kept on ice prior to use and processed within 3 h of surgery. Tonsils were washed in ice-cold Hank's balanced salt solution (HBSS; GIBCO) supplemented with 2% FCS, 5% penicillin-streptomycin solution (GIBCO), and 500 μg/mL normocin (Invivogen). Whole tonsils were partially submerged in cold, supplemented HBSS and dissected into pieces 1–5 mm in size using a sterile scalpel. Tissue fragments were separated into multiple 70 μm cell strainers and crushed using a 20 mL syringe plunger, then washed with cold, supplemented HBSS. Strained samples were pooled and passed through a 40 μm cell strainer then pelleted at 400 g for 10 min and resuspended in 15 mL cold, supplemented HBSS. Peripheral blood mononuclear cells (PBMCs) were isolated by Ficoll-Paque density gradient centrifugation. Total $CD4^+$ T cells were isolated from the PBMC fraction using the EasySep $CD4^+$ isolation kit (StemCell Technologies) and kept on ice in supplemented HBSS. Cells were immediately stained with anti-CXCR5 conjugated to either AlexaFluor 488 (BD Biosciences, 558112) or PE/Cy7 (BioLegend, 356923) depending on the downstream application, washed, and the $CXCR5^{high}$ population sorted using a FACSAria III cell sorter (BD Biosciences). Isolated Tfh cells were resuspended at 5×10^6/mL in supplemented RPMI-1640 and cultured for 3 h at 37°C, 5% CO_2 before being used for imaging. A small number of cells were retained and stained with anti-CXCR5 and anti-PD1 conjugated to AlexaFluor 488 (BioLegend, 329935) or AlexaFluor 647 (BioLegend, 329910), washed, and analyzed by flow cytometry to confirm the purity of the $CXCR5^{high}$ $PD1^+$ population.

Quantification of BTLA and HVEM Surface Expression

Isolated whole $CD4^+$ populations or isolated Tfh cells were stained with with AlexaFluor 647-conjugated anti-BTLA (BioLegend, 344520) and compared to Quantum AlexaFluor 647 MESF calibration beads (Bangs Laboratories) by flow cytometry. HVEM levels on B cells were determined by staining PBMCs from whole blood with anti-CD19-PE-Cy7 (BD Biosciences; 560911), anti-CD38-AlexaFluor 488 (BioLegend; 303511), anti-CD27-BrilliantViolet421 (BioLegend; 356417), anti-CD20-PE (BioLegend; 302305), and anti-HVEM-AlexaFluor 647 (BD Biosciences; 564411). B cell subsets were gated as follows: Activated ($CD19^+$ $CD20^+$ $CD27^+$ $CD38^+$), Memory ($CD19^+$ $CD20^+$ $CD27^+$ $CD38^-$), immature/transitional ($CD19^+$ $CD20^+$ $CD27^-$ $CD38^+$), and plasmablasts ($CD19^+$ $CD20^-$ $CD27^+$ $CD38^+$). HVEM intensity on each cell subset was converted to absolute protein numbers by reference to Quantum AlexaFluor 647 MESF calibration beads (Bangs Laboratories). Based on the mean HVEM expression on activated B cells of ~35,000/cell, minimum surface density was estimated at ~35 molecules/μm^2 assuming an upper limit of ~1000 μm^2 total area.

Supported Lipid Bilayer Preparation and Use

Supported lipid bilayers (SLB) were prepared as described previously (Choudhuri et al., 2014). Briefly, micelles of 1,2-dioleoyl-sn-glycero-3-phosphocholine (Avanti Polar Lipids Inc.) supplemented with 12.5% 1,2-dioleoyl-sn-glycero-3-[(N-(5-amino-1-carboxypentyl) iminodiacetic acid) succinyl]-Ni (Avanti Polar Lipids Inc.) were flowed onto glass coverslips hydroxylated with piranha solution, plasma cleaned, and affixed with adhesive 6-lane chambers (Ibidi). SLBs were blocked and washed, then incubated with recombinant His-tagged proteins of interest (produced in-house with the exception of HVEM-Fc-His, which was a gift from Prof. Simon Davis, University of Oxford) at the requisite concentrations to achieve the desired density: 30 molecules/μm^2 for UCHT1-FaB, 200 molecules/μm^2 for ICAM1, 100 molecules/μm^2 for CD80, 35 molecules/μm^2 for HVEM-Fc-His, and 100 molecules/μm^2 for CD40. The specific combination of unconjugated proteins or proteins conjugated to different dyes (AlexaFluors 405, 488, 568, and 657) was varied to suit the demands of each experiment. Within 2 h of preparation, SLBs were pre-warmed to 37°C and cells were infused into the SLB chambers at ~5×10^5/lane for $CD4^+$ cells or ~1×10^5/lane for Tfh cells. Samples were either incubated for 3 or 15 min at 37°C then fixed with warm 4% para-formaldehyde in PHEM buffer (60 mM PIPES, 25 mM HEPES, 10 mM EGTA, 2 mM $MgCl_2$, pH 6.9) for 10 min, or imaged live. In experiments involving labeling of transfected BTLA-SNAP-tag, cells were incubated with 0.5 μM SNAP-Cell 647-SiR ligand (New England BioLabs) in supplemented RPMI-1640 for 30 min at 37°C, washed 3 times, and incubated for a further 30 min prior to addition to SLBs.

Immunofluorescence Staining

Fixed cell samples were exposed to a second round of fixation in PHEM buffer with 2% PFA and 3% BSA (bovine serum albumin) for 10 min at room temperature in order to reduce non-specific adsorption of probing antibodies to the coverslip. Samples were washed 3 times with PHEM buffer, permeabilized with 0.1% saponin in PHEM buffer for 15 min, then washed 3 more times with PHEM buffer. Samples were then quenched with 100mM glycine for 20 min and blocked with 6% BSA in PHEM buffer for 1 h before washing 3 times with PHEM buffer. Samples were then incubated with the appropriate primary rabbit antibody (anti-SHP1, Santa Cruz Biotechnology, sc-287; anti-SHP2, Santa Cruz Biotechnology, sc-280; anti-pY493 ZAP70, Cell Signaling Technology, 2704; anti-pT538 PKCθ, Cell Signaling Technology, 9377) in PHEM buffer with 0.02% saponin, 3% BSA for 1 h. Washing was performed 3 times with PHEM buffer, 0.1% saponin, 3% BSA with 2 min between each wash, before incubating with goat anti-rabbit F(ab')2 conjugated to AlexaFluor 568 (ThermoFisher Scientific, A-21069) in PHEM buffer with 0.02% saponin, 3% BSA for 45 min. Samples

were finally washed 5 times with PHEM buffer, 0.1% saponin, 3% BSA with 2 min between each wash before imaging. A sample stained using only secondary antibody was included in each experiment as a background control.

TIRF and Confocal Imaging

TIRF imaging was performed on an Olympus cellTIRF-4Line system using a 150x (NA 1.45) oil objective. Imaging of live samples was performed at 37°C, and of fixed samples at room temperature. Confocal imaging was performed on a 37°C-controlled Olympus FV1200 confocal microscope using a 20x (NA 0.75) air objective.

Image Analysis

All image analysis was performed using the ImageJ software. Thresholded IRM images were used to define the contact area of each cell, and all pixels therein used to calculate the mean fluorescence intensity (MFI) for a given channel. Pearson colocalization coefficients (PCCs) were also calculated only for IRM-defined contact areas using the Coloc 2 plugin to perform pixel intensity correlation between channels. Identification and tracking of cells for comparison of adhesion and movement was performed using the TrackMate plugin, with total cells detected from bright field images and those forming contacts identified through IRM images. Cells were qualitatively assigned as forming synapses or kinapses based on the relative centrality and symmetry of the predominant UCHT1 and ICAM1 signals. Synapses were defined as having approximately central UCHT1 accumulations completely surrounded by a ring of ICAM1; kinapses as having a highly polarized UCHT1 accumulation with ICAM1 adjacent but not surrounding. Cells not fitting either category were defined as unassigned.

In Situ Analysis of Interaction of SHP1 and SHP2 with PD-1

Supported lipid bilayers presenting relevant ligands were prepared as described above. Biotin-CAP-phosphatidylethanolamine was used at a 0.04 molar percentage to that of dioleoylphosphatidylcholine (DOPC). Monobiotinylated Okt3 (anti-CD3 antibody, from Ebiosciences) was used at 0.5 μg/mL. The following ligands were presented on the bilayer by means of coordination chemistry between DOGS-NTA and poly-Histidine tag: 12×-His ICAM1 ectodomain at ~200 molecules/μm^2; 12×-His CD58 ectodomain at ~300 molecules/μm^2; and 12×-His PDL1 ectodomain at ~300 molecules/μm^2. Freshly isolated human $CD8^+$ T cells were electroporated with mRNA encoding PD-1 GFP or PD-1 Y248F GFP and cultured for ~8 h (Zhao et al., 2006). Cells were introduced into the flow-cells containing lipid bilayers at 37°C and fixed after 10 min by flowing in pre-warmed 2% EM-grade formic acid in PBS. SHP1/2 staining was performed as described above. Images were acquired on a Nikon Eclipse Ti inverted fluorescence microscope using an Apo TIRF 100× 1.49 NA oil objective. Diode lasers (488 nm, 561 nm and 641 nm, from Coherent) were used for TIRF illumination at the plane of bilayer. An AOTF (from Solamere Technologies) was used for choosing appropriate excitation wavelengths. A xenon arc lamp (from Newport) filtered at 490 nm was used for reflection imaging of cell contacts with the bilayer. Nikon perfect focus system® was used to achieve consistent illumination and also to compensate for axial chromatic aberration. NIS-Elements software (from Nikon) was used for hardware control of the microscope, filter and shutter wheels, AOTF, and the EMCCD camera (Andor DU-897 X-4654) during image acquisition. EMCCD camera was operated in the normal gain mode and well under saturation. Custom written macros were used for automated image analysis in ImageJ 1.44o (from NIH). Cell-boundaries were defined based on segmented ('Default' algorithm in ImageJ) reflection images. Background subtracted images were used for calculating average fluorescence intensity from each cell. PCC values between thresholded green and far-red channels ('MaxEntropy' algorithm in ImageJ) were calculated for each cell to quantitatively measure the extent of colocalization. Cluster statistics were computed using the 'Analyze Particles' routine in ImageJ after thresholding the images using the 'MaxEntropy' algorithm. Statistical significance was assessed by Mann-Whitney test using the GraphPad Prism5 software.

QUANTIFICATION AND STATISTICAL ANALYSIS

All statistical tests were done with GraphPad Prism software. The appropriate statistical test for each experiment is noted in the figures.

DATA AND CODE AVAILABILITY

Raw and processed data files for RNA sequencing analysis have been deposited in the NCBI Gene Expression Omnibus under accession number GEO: GSE130095.

Immunity, Volume *51*

Supplemental Information

The HVEM-BTLA Axis Restrains T Cell Help to Germinal Center B Cells and Functions as a Cell-Extrinsic Suppressor in Lymphomagenesis

Michelle A. Mintz, James H. Felce, Marissa Y. Chou, Viveka Mayya, Ying Xu, Jr-Wen Shui, Jinping An, Zhongmei Li, Alexander Marson, Takaharu Okada, Carl F. Ware, Mitchell Kronenberg, Michael L. Dustin, and Jason G. Cyster

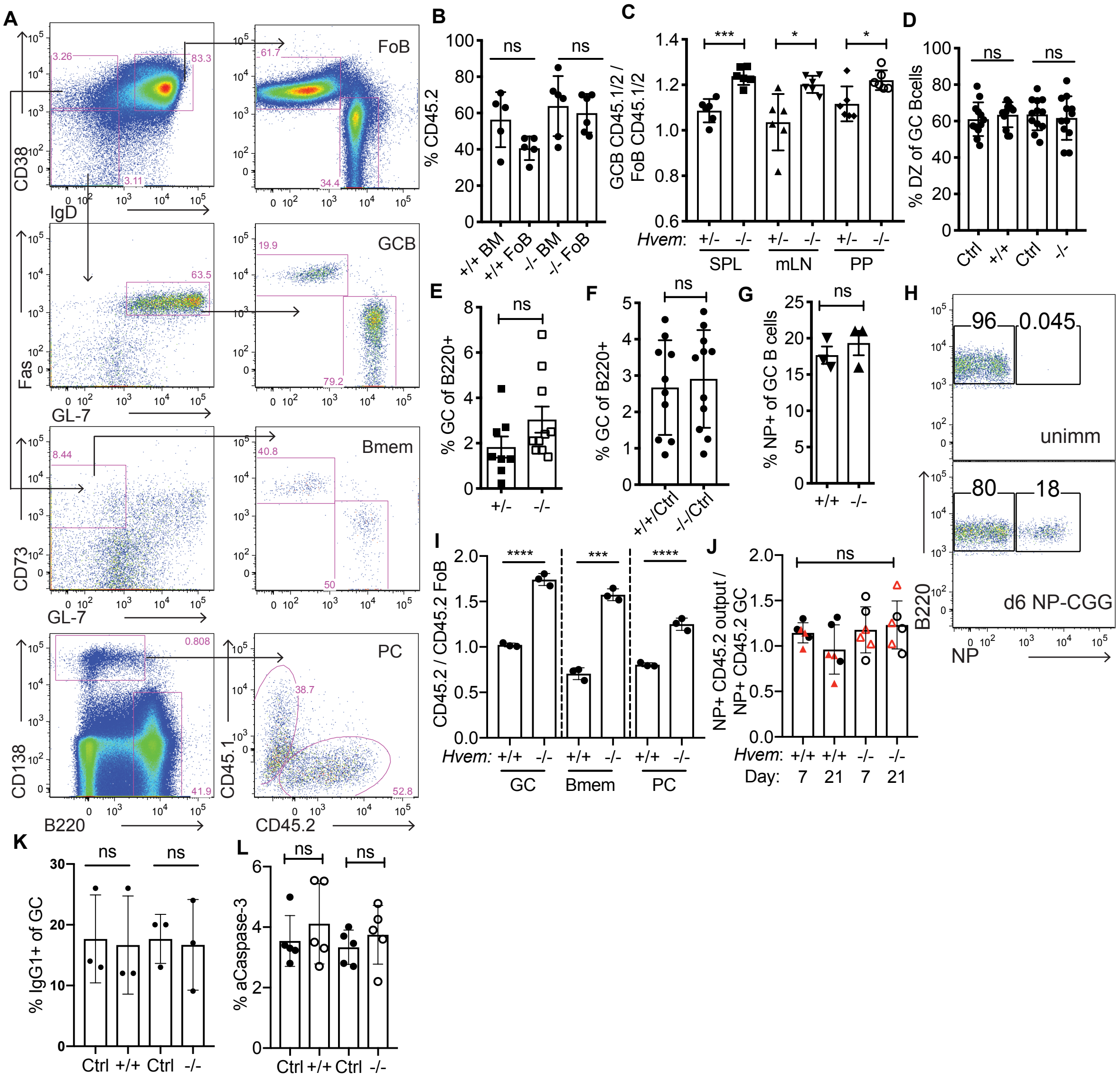

Figure S1. HVEM-deficiency increases GC B cell competitiveness in chronic GCs and GC output after an acute immune response. Related to Figure 1.

(A) Representative flow cytometric analysis of spleen Fo, GC, Bmem, and PC populations from mixed BM chimeras after SRBC immunization. (B) Contribution of CD45.2 cells to CD93$^+$ immature BM B cells and splenic Fo B cells in mixed BM chimeras. Data pooled from 2 experiments. (C) Ratio of CD45.1/2 GC to CD45.1/2 Fo B cells in mixed chimeras made with ~70% of CD45.1/2 *Hvem*$^{+/-}$ or *Hvem*$^{-/-}$ BM with ~30% CD45.1 WT BM in Spleen (SPL), mesenteric LN (mLN) and Peyer's Patches (PP), after intraperitoneal (i.p.) SRBC immunization. Data pooled from 2 independent experiments. (D) Frequency of GC B cells from mixed BM chimeras in dark zone (DZ) state (CXCR4^{high}CD86^{low}) after SRBC immunization. Data pooled from 3 experiments. (E) Frequency of GC B cells in full *Hvem*$^{+/-}$ or *Hvem*$^{-/-}$ animals 6–8 days after SRBC immunization. Data pooled from 3 experiments. (F) Frequency of GC B cells in *Hvem*$^{+/+}$ and *Hvem*$^{-/-}$ mixed BM chimeras after SRBC immunization. Data pooled from 3 experiments. (G) Frequency of NP$^+$ GC B cells in full, non-competitive *Hvem*$^{+/+}$ or *Hvem*$^{-/-}$ chimeras. (H) Representative flow cytometric analysis for splenic NP$^+$ GC B cell gating on day 6 NP-CGG alum response compared to unimmunized control. (I) Ratio of CD45.2 GC, memory B cells (Bmem), and plasma cells (PC) to CD45.2 Fo B cells at day 8 after SRBC immunization. *Hvem*$^{-/-}$ mixed chimera example shown in panel A. (J) Ratio of CD45.2 NP$^+$ splenic Bmem (black circles) or PC (red triangles) to CD45.2 NP$^+$ GC B cells at day 7 and 21 after NP-CGG immunization. Data from 1 experiment and representative of 3 experiments. (K) Frequency IgG1$^+$ of GC B cells in *Hvem*$^{-/-}$ mixed chimeras at day 8 after SRBC. (L) Frequency of anti-active Caspase-3$^+$ GC B cells from NP-CGG alum immunized mixed BM chimeras day 6-7 directly ex vivo. Data pooled from 2 experiments. 'Ctrl' refers to the respective WT CD45.1 competitor. *$P<0.05$, **$P<0.01$, ***$P<0.001$, ****$P<0.0001$. Unpaired two-tailed Student's t test (B-G, I, K-L), Ordinary One-Way Anova with Bonferroni's multiple comparisons test (J).

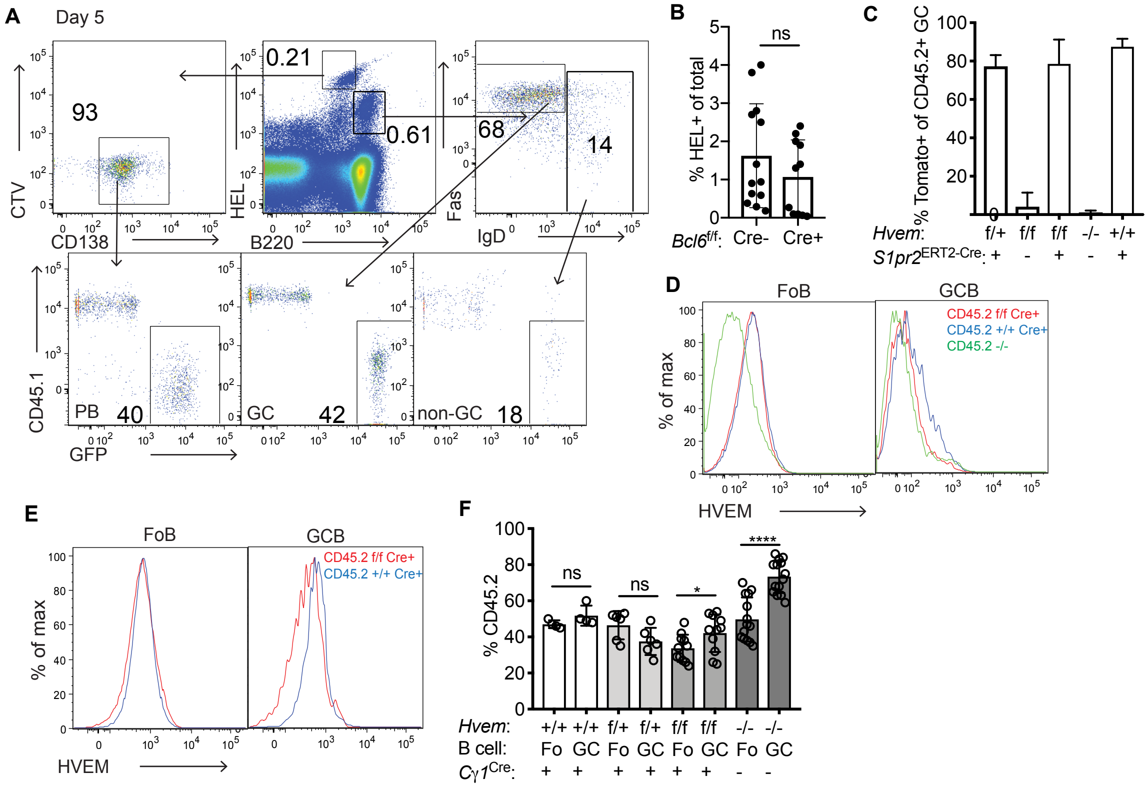

Figure S2. HVEM-deficiency provides B cells with a proliferation advantage early in the response and within the GC. Related to Figure 2.

(A) Representative flow cytometric analysis for gating GFP⁺ HEL⁺ B cells as HEL-intracellular-high CD138⁺ plasmablasts (PB) and HEL-intermediate IgD^{lo}Fas⁺ GC B cells at days 4.5–5. (B) Frequency of HEL⁺ cells of total spleen in *Bcl6*$^{f/f}$ *Cd4*^{Cre} or controls at day 5–7 after 2x-HEL-SRBC. Data pooled from 3 experiments. (C) Frequency of tdTomato⁺ of CD45.2 GC B cells after tamoxifen treatment. Data are pooled from 3 experiments. (D) Representative flow cytometric analysis of HVEM surface levels on Fo and GC B cells in CD45.2 compartment. Red *Hvem*$^{f/f}$ *S1pr2*^{ERT2Cre}, blue *Hvem*$^{+/+}$ *S1pr2*^{ERT2Cre}, and green *Hvem*$^{-/-}$. (E) Representative flow cytometric analysis of HVEM surface levels in *Hvem*$^{f/f}$ *Cγ1*^{Cre} Fo and GC B cells in red and *Hvem*$^{+/+}$ *Cγ1*^{Cre} in blue. (F) *Hvem*$^{f/f}$ *Cγ1*^{Cre} and respective control CD45.2 mixed BM chimeras immunized with NP-CGG and analyzed day 10–13 for the frequency of CD45.2 cells in the Fo and GC compartments. Data are pooled from 3 experiments. *P<0.05, ***P<0.001, ****P<0.0001. Unpaired two-tailed Student's t test (B, F).

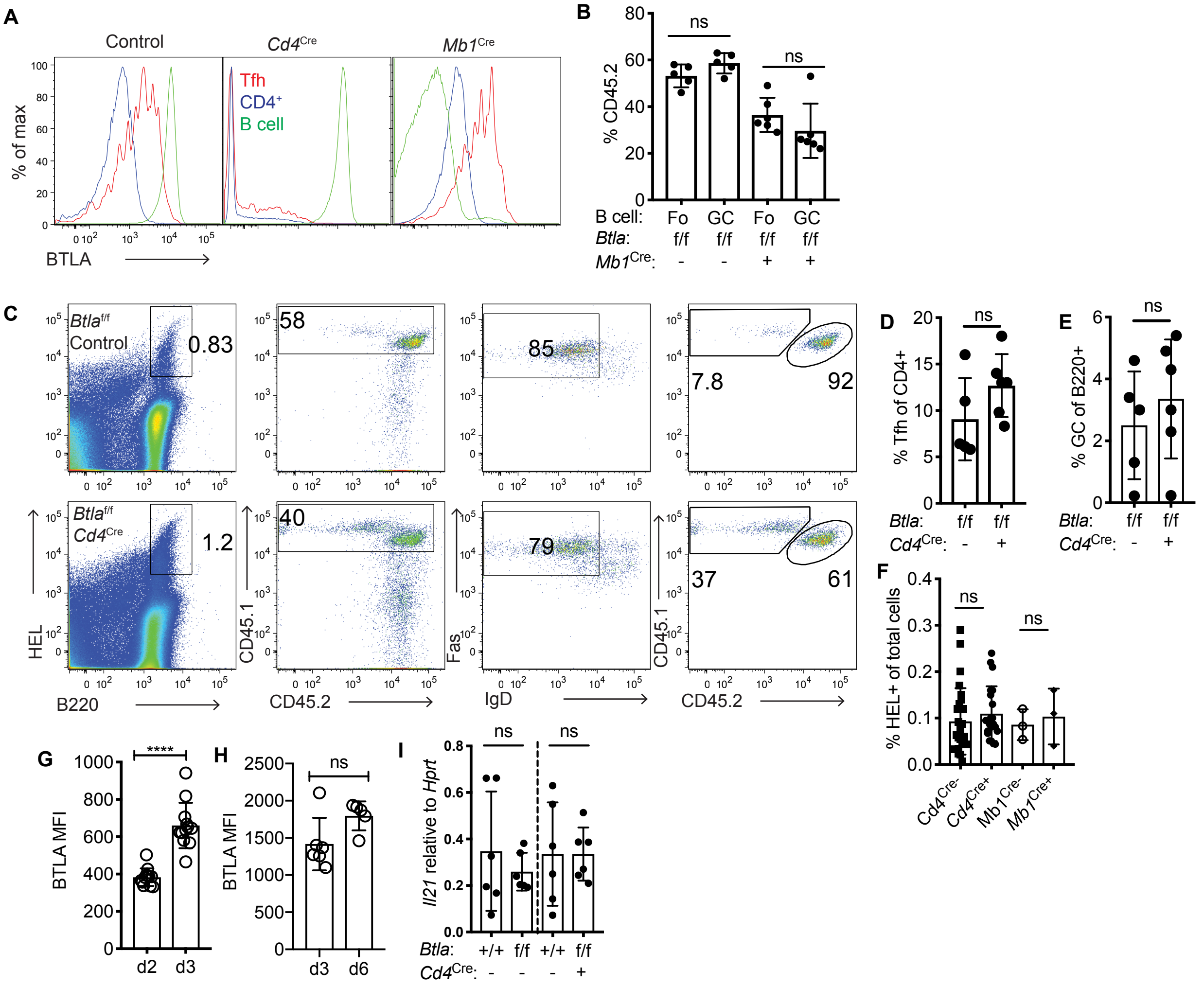

Figure S3. Conditional deletion of BTLA in T cells abrogates HVEM-deficient GC advantage. Related to Figure 4.

(A) BTLA MFI B220^{+} B cells (green), CD4^{+}TCRβ^{+}CXCR5^{+}PD-1^{+} Tfh cells (red), and CD4^{+}TCRβ^{+}CXCR5^{-}PD1^{-} non-Tfh CD4^{+} T cells (blue) in *Btla*$^{f/f}$ animals. Cre^{-} left, *Cd4*^{Cre} middle, and *Mb1*^{Cre} right. (B) Contribution of CD45.2 *Btla*$^{f/f}$ control or BTLA-B cell-deficient *Btla*$^{f/f}$ *Mb1*^{Cre} CD45.2 cells to Fo and GC populations in spleen of mixed BM chimeras made with ~50% CD45.2 and ~50% WT CD45.1 BM at day 7 after SRBC immunization. Data are pooled from 2 experiments. (C) Representative flow cytometric analysis of the frequency of *Hvem*$^{-/-}$ CD45.1/2 HEL^{+} GC B cells in *Btla*$^{f/f}$ control (top) and *Cd4*^{Cre} (bottom) recipients at day 6 after 2x-HEL-SRBC. (D) Frequency of Tfh cells of CD4^{+} T cells in *Btla*$^{f/f}$ *Cd4*^{Cre} animals in the spleen after SRBC. Data are pooled from 2 experiments. (E) Frequency of GC B cells of B cells in *Btla*$^{f/f}$ *Cd4*^{Cre} animals in the spleen after SRBC. Data are pooled from 2 experiments. (F) Frequency of HEL^{+} B cells of total splenocytes after Hy10 transfer and 2x-HEL-SRBC immunization. Data are pooled from 4 experiments. (G) BTLA MFI on OT-II T cells at 48 hr and 72 hr after HEL-OVA or DEL-OVA immunization. Data are pooled from 2 experiments. (H) BTLA MFI on Tfh cells at 3 days and 6 days after 2x-HEL-SRBC immunization. (I) *Il21* mRNA transcript relative to *Hprt* from sorted splenic Tfh cells from *Btla*$^{f/f}$ control CD45.2 or *Cd4*^{Cre} mixed CD45.1/2 WT BM chimeras at day 7 SRBC immunization. Data are pooled from 2 experiments. ****$P<0.0001$. Unpaired two-tailed Student's t test (B, D-I).

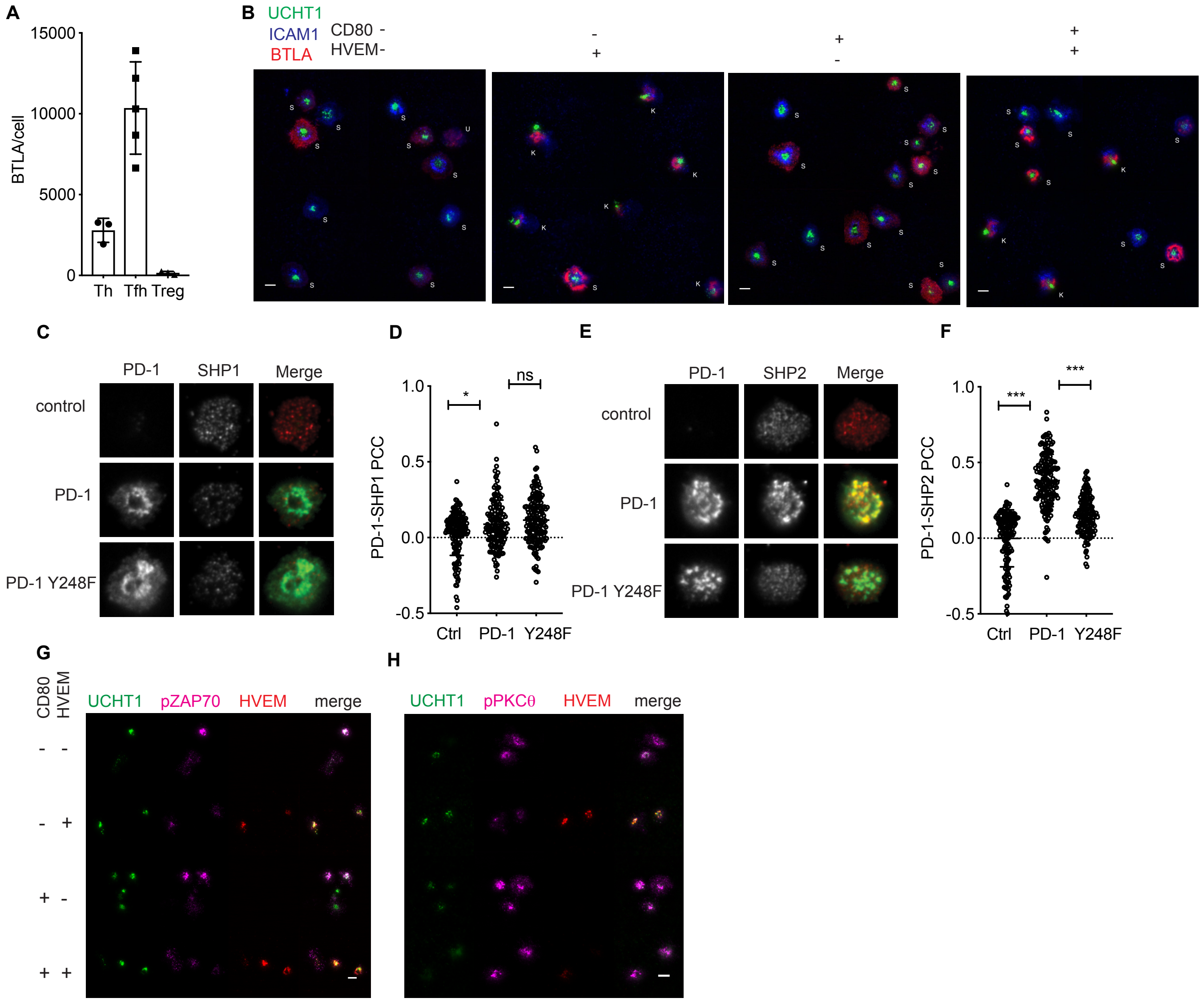

Figure S4. BTLA-HVEM at the immunological synapse recruits SHP1 to inhibit signaling in Tfh cells. Related to Figure 5.

(A) Expression of BTLA on human tonsil Tfh cells, Treg cells and untransfected CD4$^+$ blasts (Th). (B) Representative images of synapse (s) or kinapse (k) states in BTLA-transfected human CD4$^+$ T cell blasts on supported lipid bilayers containing anti-CD3 (UCHT1), ICAM1 and/or HVEM, CD80 as indicated. (C) Representative images of SHP1 recruitment to the synapse in relation to PD-1 or mutant PD-1 Y248F. (D) Quantification of SHP1 colocalization with WT PD-1 or mutant Y248F with or without (Ctrl) PDL-1 in the bilayer. (E) Representative images of SHP2 recruitment to the synapse in relation to PD-1 or mutant PD-1 Y248F. (F) Quantification of SHP2 colocalization with PD-1 or mutant Y248F with or without (Ctrl) PDL-1 in the bilayer. (G) Representative images of p-ZAP70 and (H) p-PKCθ in human CD4$^+$ T cell blasts transfected with BTLA shown at the same LUT. Scale bars are 5 μm. *P<0.05, **P<0.01, ***<P0.001, ****P<0.0001. Mann-Whitney test (D, F.)

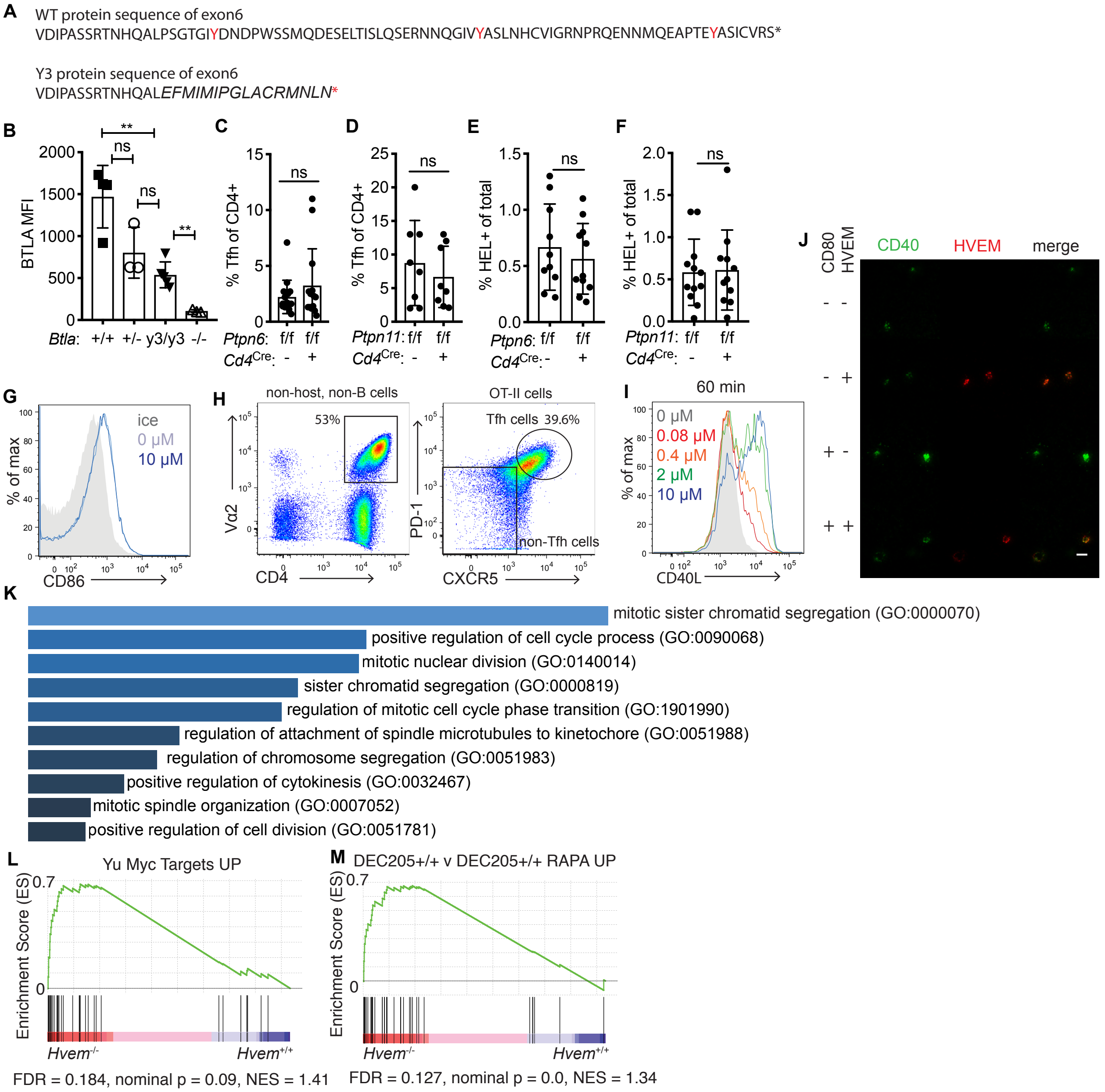

Figure S5. BTLA signaling into the T cell through SHP1 is required for HVEM-deficient GC B cell competitiveness. Related to Figure 6.
(A) Protein sequence of exon 6 of WT BTLA and mutant BTLA Y3. A short out-of-frame deletion was introduced at position 38 of exon 6 using CRISPR mutagenesis in the mouse embryo. Italics indicates nonsense sequence occurring after the deletion. * denotes stop codon. (B) BTLA expression on *Btla*$^{+/+}$, *Btla*$^{+/-}$, *Btlay*$^{3/y3}$, *Btla*$^{-/-}$ Tfh cells. Data pooled from 2 experiments. (C, D) Frequency of Tfh cells in *Ptpn6*$^{f/f}$ (SHP1) (C) and *Ptpn11*$^{f/f}$ (SHP2) (D) control and *Cd4*^{Cre} animals. Data pooled from 2 experiments. (E, F) Frequency of HEL^{+} cells in *Ptpn6*$^{f/f}$ (E) and *Ptpn11*$^{f/f}$ (F) control and *Cd4*^{Cre} animals. Data pooled from 2 experiments. (G) Representative flow cytometric analysis of CD86 surface expression on B cells after a 2 hr pulse with or without OVA peptide (10 μM) and incubation with OT-II Tfh cells for 30 min compared to B cells kept on ice directly ex vivo. (H) Gating strategy for OT-II Tfh cells at d3 after HEL-OVA-SRBC + poly-I:C. (I) Representative flow cytometric analysis of CD40L surface mobilization on OT-II Tfh cells after a 60 min incubation with B cells pulsed with a titration of OVA peptide (0-10 μM). (J) Representative images of CD40 recruitment to the immunological synapse with or without HVEM and CD80 present in the lipid bilayer after 15 min incubation with blasting human CD4^{+} T cells with standardized LUT across panels so fluorescence can be directly compared. Scale bars are 5 μm. (K) Gene ontology (GO) biological processes of genes significantly (padj <0.01) upregulated in *Hvem*$^{+/+}$ v *Hvem*$^{-/-}$ GC B cells at d11 after NP-CGG from RNA-sequencing through Enrichr. Sorted by p-value. (L) GSEA of differentially expressed genes from RNA-sequencing of *Hvem*$^{+/+}$ v *Hvem*$^{-/-}$ mixed BM chimeras at d11 of the NP-CGG alum response compared to Yu Myc targets UP gene set. (M) GSEA of differentially expressed genes from RNA-sequencing of *Hvem*$^{+/+}$ v *Hvem*$^{-/-}$ mixed BM chimeras at d11 of the NP-CGG alum response compared to significant genes (<0.01 padj) with >2 fold increased gene expression in DEC205-WT GC B cells 24 hr after anti-DEC205-OVA compared to DEC205-WT Rapamycin treated. *P<0.05, **P<0.01, ***<P0.001, ****P<0.0001. Unpaired two-tailed Student's test (B-F).
